# Comparing Long-Read Assemblers to Explore the Potential of a Sustainable Low-Cost, Low-Infrastructure Approach to Sequence Antimicrobial Resistant Bacteria With Oxford Nanopore Sequencing

**DOI:** 10.3389/fmicb.2022.796465

**Published:** 2022-03-03

**Authors:** Ian Boostrom, Edward A. R. Portal, Owen B. Spiller, Timothy R. Walsh, Kirsty Sands

**Affiliations:** ^1^Division of Infection and Immunity, Department of Medical Microbiology, Cardiff University, Cardiff, United Kingdom; ^2^Department of Zoology, Ineos Oxford Institute for Antimicrobial Research, University of Oxford, Oxford, United Kingdom

**Keywords:** antimicrobial resistance (AMR), Oxford Nanopore Technology (ONT), long-read sequencing (LRS), antimicrobial resistance genes (ARG), Guppy, MinION, plasmid, *de novo* assembly

## Abstract

Long-read sequencing (LRS) can resolve repetitive regions, a limitation of short read (SR) data. Reduced cost and instrument size has led to a steady increase in LRS across diagnostics and research. Here, we re-basecalled FAST5 data sequenced between 2018 and 2021 and analyzed the data in relation to gDNA across a large dataset (*n* = 200) spanning a wide GC content (25–67%). We examined whether re-basecalled data would improve the hybrid assembly, and, for a smaller cohort, compared long read (LR) assemblies in the context of antimicrobial resistance (AMR) genes and mobile genetic elements. We included a cost analysis when comparing SR and LR instruments. We compared the R9 and R10 chemistries and reported not only a larger yield but increased read quality with R9 flow cells. There were often discrepancies with ARG presence/absence and/or variant detection in LR assemblies. Flye-based assemblies were generally efficient at detecting the presence of ARG on both the chromosome and plasmids. Raven performed more quickly but inconsistently recovered small plasmids, notably a ∼15-kb Col-like plasmid harboring *bla*_*KPC*_. Canu assemblies were the most fragmented, with genome sizes larger than expected. LR assemblies failed to consistently determine multiple copies of the same ARG as identified by the Unicycler reference. Even with improvements to ONT chemistry and basecalling, long-read assemblies can lead to misinterpretation of data. If LR data are currently being relied upon, it is necessary to perform multiple assemblies, although this is resource (computing) intensive and not yet readily available/useable.

## Introduction

Antimicrobial resistance (AMR) is a serious global threat with the WHO identifying that a “post-antibiotic era—in which common infections and minor injuries can kill—far from being an apocalyptic fantasy, is instead a very real possibility for the twenty-first Century” (WHO Antimicrobial Resistance Division, 2014). AMR accounts for 700,000–750,000 annual deaths worldwide (DAG Hammarskjöld Foundation, 2019; IAGC, 2019). The Review on Antimicrobial Resistance (O’Neill, 2016) estimated the global burden of AMR to be 10 million deaths by 2050, an estimate provided well before the uptick seen during COVID-19 (Lai et al., 2021). A combination of inappropriate antibiotic usage (both clinical and veterinary), poor antimicrobial stewardship, and a lack of appropriate and timely antibiotics drives the spread of AMR, particularly *via* exchange of mobile genetic elements (MGE), the AMR mobilome.

Currently, complete genomic characterization requires both short-read (SR) and long-read sequencing (LRS) technologies. The PacBio platform can offer fully circularized chromosomal and plasmid DNA with high fidelity (HiFi) reads; however, access to this 354-kg instrument and the equipment/services to prepare large yields of high-quality DNA are obvious limitations. PacBio and Illumina platforms have challenging equipment requirements, and both require accounting for and adapting to GC bias. Oxford Nanopore Technologies (ONT) platforms are considerably smaller with fewer laboratory requirements, offer “on the spot” analysis, and reduce GC bias, but have historically suffered from low read accuracy and stochastic read-depth dependent errors (Chen et al., 2021; Vasiljevic et al., 2021).

A PubMed search (September 18, 2021) revealed 84 hits for “Oxford Nanopore” + “antimicrobial resistance,” with over 70 published in the last 3 years. Many used ONT to sequence a specific isolate/cohort of isolates to complement existing SR to resolve MGE associated with AMR (Yu et al., 2019; Chan et al., 2020; Diaconu et al., 2020; Faccone et al., 2020; Sadek et al., 2020).

ONT has been invaluable for direct field sequencing during the Ebola (Quick et al., 2016) and Zika outbreaks (de Jesus et al., 2019). While there are differences between bacterial and viral WGS, notably the size and complexity of the bacterial accessory genome, the increase in publications featuring bacterial AMR WGS solely from LR ONT sequencing highlight increased interest (Juma et al., 2021; Tegha et al., 2021). There are now several options for LR assembly/polishing (Chen et al., 2020; Wick and Holt, 2021). The release of new software including Trycycler, which aims to generate a consensus LR assembly from several iterations at input (Wick et al., 2021a), will further encourage the use of LR data.

Resources for identifying and monitoring infectious disease outbreaks are finite, particularly in low- and middle-income countries (LMICs). Purchasing sequencing platforms that require highly controlled laboratory environments is often not viable. Although the COVID-19 pandemic has led to a favorable re-evaluation of the merit in active genomic surveillance, limited resources in LMICs necessitate particular care when identifying processes/infrastructure to implement and support. While the aforementioned practicalities are not necessarily mentioned, a salient number of publications during the last 12 months promote successful “in house” Oxford Nanopore sequencing in African LMICs (Juma et al., 2021; Tegha et al., 2021).

Our group is interested in the feasibility, reproducibility, and cost-effectiveness of infrastructure-light identification of antibiotic resistance genes using ONT. We will (1) look at whether ONT’s rapidly reiterating raw-signal analysis supports further expansion in LR sequencing, (2) compare methods using ONT’s R9 or R10 membrane proteins, (3) compare LR and hybrid assemblies, (4) analyze LR assemblies with a focus on ARG detection, and (5) define the relative infrastructure and running costs of maintaining on-demand nanopore sequencing, all of which will be considered in the context of (6) whether LRS is desirable for resource-limited environments.

## Materials and Methods

### Bacterial Culture and gDNA Extraction

Bacterial isolates were plated from frozen stocks onto selective and differential agar (using antimicrobial susceptibility data) and incubated overnight at 37°C. Three gDNA extraction methods were performed depending upon bacterial species and extraction date. Isolates cultured between 2018 and 2021 were included and pseudoanonymized. The chloroform precipitation with CTAB method was performed between 2018 and 2019, and details of the method are in Supplementary Material. For two different Qiagen-based gDNA extractions, bacterial isolates were cultured into liquid broth [LB for Gram-negative bacteria (GNB) and Staphylococci, Todd Hewitt for Streptococci, and mycoplasma/ureaplasma selective media for Mollicutes]. Liquid cultures were centrifuged to produce a bacterial pellet (Supplementary Methods in the Supplementary Material details centrifugation and pre-treatment steps) and loaded on the Qiacube (for spin column-based extraction) or the Qiagen EZ1 Advance XL (for magnetic bead-based extraction) (Qiagen, Germany).

gDNA was quantified using the dsDNA BR assay kit on a Qubit fluorometer 3.0 or 4.0 and kept at 4–8 or −20°C for chloroform precipitated gDNA and Qiagen extracts, respectively.

### Whole-Genome Sequencing

For Illumina MiSeq, genomic libraries were prepared using Nextera XT V2 (Illumina, United States), with bead-based normalization. Paired-end WGS was performed using the V3 kit. Each sequencing run was multiplexed up to 48 isolates to provide > 20X coverage.

For ONT, gDNA (Qiagen) was concentrated and purified at a 1:1 ratio using SPRI beads (Mag-Bind TotalPure; Omega) with 15 μl water elution. Genomic libraries were prepared using the Rapid Barcoding Kit (SQK-RBK004; ONT) and sequenced on a FLO-MIN106 R9.4 flow cell using a MinION (ONT). Sequencing was performed on single-use MinION flow cells for a running time of 72 h with default parameters within MinKNOW unless otherwise specified.

We compared yield and LRS metrics between R9 and R10 flow cells. gDNA (*n* = 53) was extracted using the Qiacube, concentrated using SPRI beads, and libraries generated using the 96-Rapid Barcoding Kit (SQK-RBK110.96; ONT). gDNA was pooled and divided into 6 aliquots for simultaneous flow cell loading onto three flow cells; two R10 (one on a MinION Mk1B connected to an Intel i7-8750H laptop, another on a MinION Mk1C) and one R9 (Mk1B connected to an Intel i7-6700 desktop computer). Following the initial sequencing period, flow cells were washed (WSH003), QC checked to determine recovery of nanopores, and re-loaded with the remaining aliquots of gDNA (stored at 4°C).

### Bioinformatics Analysis

#### Illumina MiSeq Sequencing Data: QC of Input Reads

Several studies have shown that hybrid *de novo* assemblies significantly improve the quality of contiguous chromosome and plasmid assembly (Wick et al., 2017; Lipworth et al., 2020), and therefore this comparison was not the primary aim. Here, we trimmed Illumina barcodes and applied QC trimming (–phred33 -q 25) with Trimgalore (v0.5.0) (Krueger, 2018). Trimmed fastq were assembled into contigs for SR analysis using shovill (v0.9.0) (Seemann, 2018) and were also input into Unicycler (v0.4.7 and v0.4.9) (Wick et al., 2017) assemblies. Seqfu (v1.3.1) (Telatin et al., 2021) was used to count the number of reads (paired-end aware). Isolates were excluded from the dataset if SR were insufficient for > 1x of the genome [number of reads × length of reads (∼270)/length of genome], in addition to insufficient LR. Further exclusions were based on sequencing read and assembly metrics.

#### Basecalling Fast5 Files and Demultiplexing

Basecalling was performed twice for each isolate. Initial basecalling used Guppy (v2.1.3, v3.2.10, v3.6.5, v4.2.5, and v4.5.4) within MinKNOW. Archived FAST5 were re-basecalled using Guppy v5.0.11 and NVIDIA V100 GPUs. Data in the format of 4,000 FAST5 reads per sub-directory (2018 data) were pre-processed with the ont_fast5_api converter single_to_multi_fast5 parameter. All original LR were demultiplexed using Porechop (v0.2.4) (Wick, 2018b). For all re-basecalled LR, Guppy –trim_barcodes parameter was applied.

#### QC for Oxford Nanopore Technology Sequencing Data

NanoPlot (v1.19.0) (De Coster et al., 2018) was used to generate LRS metrics including N50, number of reads, and mean read quality (Q score). LRS trimming programs Filtlong (v0.2.0) (Wick, 2018a) (filtlong –min_length 1,000 –keep_percent 90) and Nanofilt (v2.6.0) (De Coster et al., 2018) (Nanofilt -q 10 –l 1,000 –headcrop 50) were used to create different LR assemblies as described later.

#### Hybrid Assembly: Illumina MiSeq and Oxford Nanopore Technology

For all 200 isolates, the original LR were assembled with corresponding SR using Unicycler (v0.4.7) (Wick et al., 2017). A subset (*n* = 62/200) were hybrid reassembled in Unicycler using LR basecalled with Guppy v5.0.11 and trimmed with Filtlong as described earlier.

#### Long Read Assembly Comparison

**FIGURE 1:**
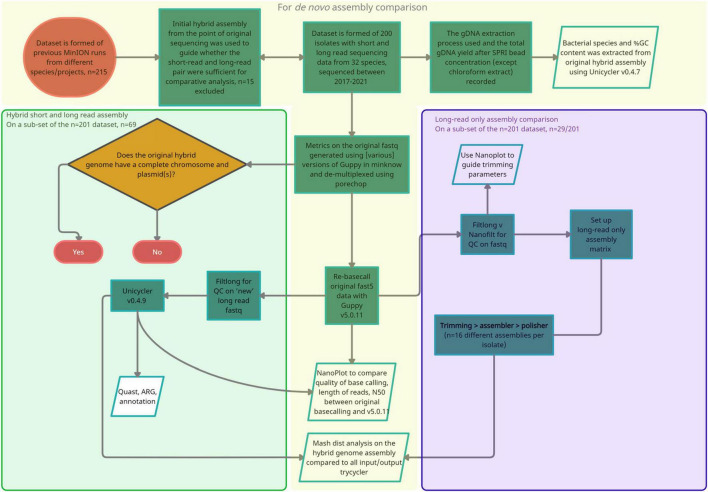
A flow diagram summarizing the approach and methods taken for the three stages of analysis. (1) Orange, Isolate selection, re-basecalling with Guppy v.5.0.11 and comparison of long read metrics, (2) green, Repeat hybrid assembly with Unicycler v0.4.9 and re-basecalled long reads as input (–1 parameter), (3) purple, Additional long-read only assembly comparison.

**FIGURE 2:**
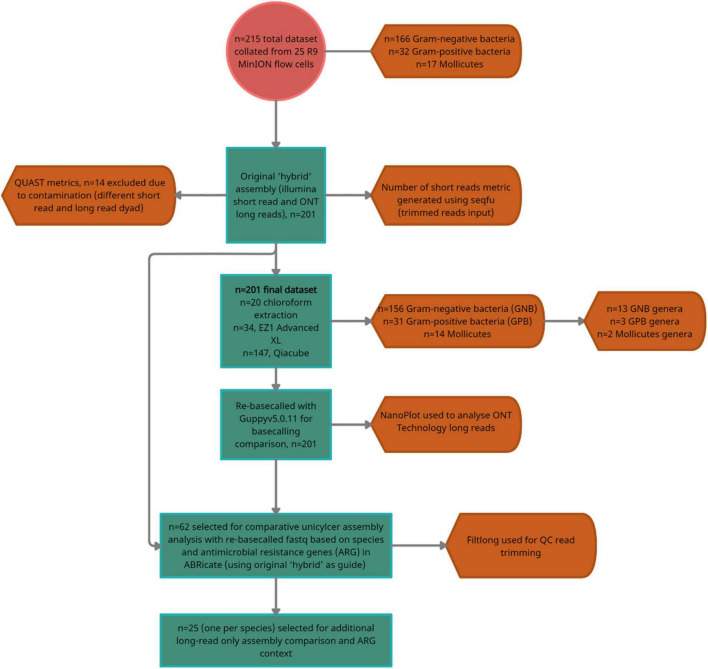
A flow diagram summarizing the dataset, number of exclusions and the number of isolates selected for the two-stage downstream analyses; comparative hybrid assembly (Unicycler) with re-basecalled (Guppy v5.0. 11) fastq (*n* = 62/200), and additional comparison with multiple long read assemblers (*n* = 25/200).

A subset of isolates (*n* = 25/200) were selected to evaluate differences in basecalling and *de novo* assembly quality, with LR assemblers compared with hybrid assembly as outlined in Figures 1, 2. For continuity, isolates included in this analysis were also selected for additional Unicycler hybrid assembly with the latest basecalled LR using Guppy (v5.0.11). The combination of sequence read trimming programs [Filtlong (v0.2.0) and Nanofilt (v2.6.0)], LR assemblers [Canu (v2.1.1), Flye (v2.8.1), Raven (v1.5.1), and Miniasm (operated within Unicycler v0.4.9)], and LR assembly polishers is listed in Supplementary Figure 1. Racon (v1.3.1) (Vaser et al., 2017) was not required for Flye assemblies before medaka but was performed on Raven and Miniasm (within Unicycler -l parameter) as default, and a single round for Canu assemblies using BWA v0.7.17 (Li and Durbin, 2009) and Samtools v1.10 (Li et al., 2009) to map reads to the draft contigs. Medaka_consensus was performed on all assemblies. Default parameters were used throughout except relaxed minimum coverage as required for assembly comparisons. For Miniasm within Unicycler, the –mode conservative was used.

#### Genome Analysis

Quast (v5.0.2) (Gurevich et al., 2013), ABRicate (v0.9.7) (Seemann, 2019a), and mlst (v2.17.6) (Seemann, 2019b) were used to assess assembly quality, identify ARG and/or MGE, and sequence type (ST), respectively. Resfinder (v4.1) (Bortolaia et al., 2020) and mobile element finder (Johansson et al., 2020) (v1.0.2) databases were used within ABRicate. Genes were detected at 100% coverage and 100% ID unless otherwise stated. Qualimap (v2.2.1) (Okonechnikov et al., 2016) bamqc was performed on the BAM files produced during the Canu-Racon assemblies to assess mean sequencing coverage for the LR. Assemblies were annotated using Prokka (v1.15.5) (Seemann, 2014). Bandage (v0.8.1) (Wick et al., 2015) was used to extract the contig/plasmid of interest and examined for sequence similarity and annotation in Geneious (2020 v1.2). Mash genome distance estimation (v2.3) (Ondov et al., 2016) was performed on all assemblies from the same isolate using the hybrid assembly as reference. GSAlign v1.0.22 (Lin and Hsu, 2020) (-sen, sensitive mode) was used to assess sequence alignment between long-read assemblies and Unicycler reference.

#### R9 v R10 Sequence Read Analysis

Six FAST5 directories (2 per flow cell) were uploaded to a HPC (high-performance computer) for GPU basecalling using Guppy v5.0.11. Basecalling was performed per flow cell generating three sets of reads per isolate. Nanoplot (v1.19.0) was performed individually and on the concatenation of the reads to one fastq file.

### Cost Analysis and Computing Requirements

Our cost analysis incorporates pricing from (1) a centralized catalog incorporating over 80 UK/EU suppliers with Higher Education pricing agreements (Cardiff University, 2021), (2) LMIC-specific pricing where suppliers and manufacturers were willing to negotiate on this point, and (3) not-for-profit research pricing applied to our laboratories’ online catalog accounts. Start-up costs were calculated for equipment and consumables capable of generating 48 isolates, the least common denominator among kits. Staffing, electrical, shipping, and waste disposal costs were excluded as being too variable between LMIC collaborators to incorporate in this analysis.

## Results

### Dataset Selection, Species Diversity, and Antimicrobial Resistance Genes

**FIGURE 3:**
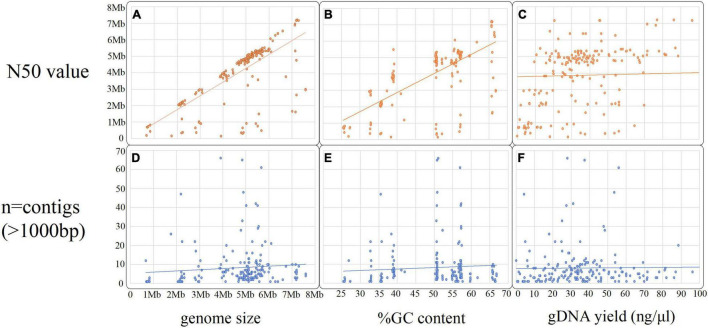
Scatter plots summarizing the hybrid assembly metrics and gDNA extraction yield (ng/μl), **(A–C)** compares the N50 value against the total genome size, the %GC content and gDNA yield input and graphs **(D–F)** compares the number of contigs (over 1,000 bp) against the same three variables.

In total, *n* = 215 isolates with SR and LR were selected (Figure 2). Fifteen isolates were excluded as they contained > 100 contigs, were contaminated, and/or SR and LR data differed (sequencing was largely performed 1–6 months apart, often, with different gDNA). The dataset (*n* = 200) (Figure 2 and Table 1) represents 30 species ranging 0.7–8 Mb (Figures 3A,D) spanning a wide GC content (25–67%) (Figures 3B,E). For *n* = 167/200, the genome size and assembly N50 were similar (Figure 3A), and 17 isolates had an N50 value less than 1 Mb (Figure 3A). For *n* = 124/200 isolates, the chromosome was complete; for *n* = 142/200, at least one plasmid was circularized (Supplementary Table 1). Chloroform extraction generated the highest overall gDNA yields, between ∼1 and > 100 ng/μl (Figures 3C,F). All isolates had fewer than 70 contigs and *n* = 180/200 had fewer than 20 contigs (Figure 3) (median *n* = 5, mean *n* = 8).

**TABLE 1:** The bacterial species included in the dataset for analysis (*n* = 32 different species), the number of each species, and the approximate %GC content based on the (original basecalled *de novo* hybrid assembly).

Species	*n* =	%GC
*Ureaplasma parvum*	6	25
*Ureaplasma urealyticum*	4	25
*Mycoplasma hominis*	4	31
*Staphylococcus aureus*	9	32
*Staphylococcus sciuri*	2	32
*Enterococcus faecalis*	1	34
*Streptococcus agalactiae*	17	35
*Streptococcus pyogenes*	1	38
*Acinetobacter baumannii*	14	39
*Acinetobacter* sp.	4	39
*Streptococcus sinensis*	1	42
*Escherichia coli*	45	50
*Morganella morganii*	1	51
*Salmonella enterica*	4	52
Unclassified Enterobacterales	1	53
*Enterobacter asburiae*	1	55
*Enterobacter cloacae*	3	55
*Enterobacter hormaechei*	1	55
*Enterobacter kobei*	4	55
*Klebsiella michiganensis*	1	55
*Klebsiella oxytoca*	1	55
*Leclercia adecarboxylata*	1	55
*Raoultella ornithinolytica*	1	55
*Alcaligenes faecalis*	1	56
*Enterobacter bugandensis*	2	56
*Enterobacter chengduensis*	1	56
*Klebsiella pneumoniae*	40	57
*Klebsiella quasipneumoniae*	1	57
*Serratia marcescens*	5	59
*Pseudomonas putida*	1	61
*Pseudomonas aeruginosa*	20	66
*Burkholderia cenocepacia*	2	67
Total	200	

Isolates for inclusion were (1) GNB species with focus to extended spectrum β-lactamase (ESBL) genes, carbapenemase genes, mobile colistin resistance genes, and mobile tigecycline resistance genes; (2) Gram-positive bacterial species including *Staphylococci* with *bla*_*MecA*_ and Streptococci with tetracycline and macrolide AMR genes; and (3) Mollicutes with point mutations causing resistance to macrolides (e.g., erythromycin). Supplementary Figure 2 summarizes the (acquired) ARG detected from the original hybrid *de novo* assemblies. Carbapenemase ARGs were detected in *n* = 69, mobile colistin resistance (mcr) genes in *n* = 17, and mobile tigecycline ARGs *tet*(X) in *n* = 13. Aminoglycoside, b-lactam, and tetracycline resistance genes were commonly found. In total, 2,045 ARG were detected within this dataset and through matching up ARG-plasmid *via* LR assembly, 890 ARG were located on the chromosome and 1,155 on plasmids (Supplementary Figure 2).

### Comparison of Long-Read Sequences: Re-basecall?

**FIGURE 4:**
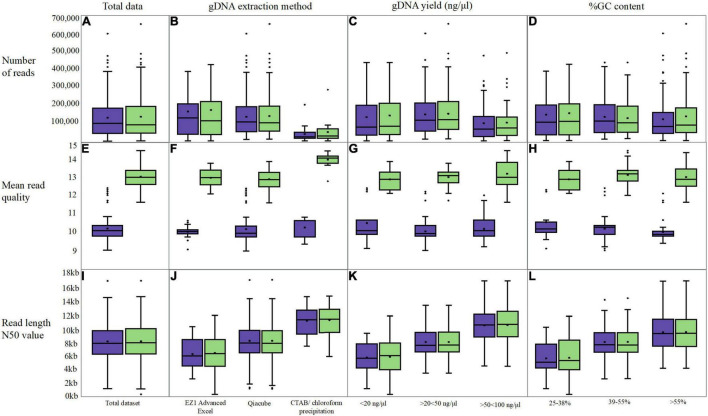
Box and whisker plot s comparing original fastq metrics (purple) with FAST5 reads re-basecalled with Guppy v5.0.11 (green). Three long-read metrics were assessed; number of reads **(A–D)**, the mean read quality **(E–H)** and the read length N50 value **(I–L)** In addition to assessing overall read metrics following re-basecalling **(A,C,I)**, data was grouped into categories for gDNA extraction **(B,F,J)**, the gDNA yield **(C,G,K)** and the %GC content **(D,H,L)**.

The average number of reads generated from the original basecalled data was 132,790 (Supplementary Table 2) and this increased slightly to 138,246 (Figure 4A) post Guppy v5.0.11 with *n* = 141/200 showing > 1% increase (an average increase for the *n* = 141/200 of 12%). For *n* = 16/200, the percent increase in the number of reads was between 30 and 50%, and this was noted for mainly *E. coli* and *K. pneumoniae* isolates extracted using either the QIAcube platform or the chloroform precipitation across three flow cells sequenced in 2019. An isolate of *M. hominis* had a 49% increase in the number of reads following Guppy v5.0.11 basecalling; however, the N50 value dropped markedly from 4,666 to 176 bp. Although limited by variable extraction category sample sizes, there was little difference when analyzing the number of basecalled reads according to gDNA yield and GC content (Figures 4C,D). We did, however, notice a reduction in the number of reads between 40 and 70% for 12 *E. coli* isolates sequenced in June 2019. These were sequenced in the same experiment and therefore likely represent a lower quality sequencing library input. A *Streptococcus* with the largest reduction (from 141,365 to 42,568 LR) also had indicators of poor-quality input gDNA (a mean Q-score increasing from 10.1 to 12.4, and a low N50 which increased from 3,767 to 3,841 bp).

The fewest reads were generated when CTAB/chloroform extraction was performed (Figure 4B); however, the length of the reads (N50) was greater at ∼12 kb compared with 8.2 and 6.4 kb for the QIAcube and EZ1 Advanced XL platforms, respectively. A smaller mean N50 value was obtained for those isolates extracted using the EZI Advanced XL platform (∼6.4 kb); however, this method was exclusively used for (*n* = 34/200) Mollicutes and *Streptococci/Enterococci* species (with bead beating prior to loading), which have the smallest genomes. The QIAcube automated platform was used for the majority of gDNA extractions from 24 different species of isolates in this dataset (*n* = 146/200). Although the N50 values were markedly higher for the isolates extracted using the CTAB/chloroform extraction (Figure 4J), this extraction was discontinued in 2019 to curtail risks and reduce labor requirements; therefore, this dataset only includes *E. coli* and *K. pneumoniae* isolates (*n* = 20/200).

Comparing the read N50 values post Guppy v5.0.11 re-basecalling, *n* = 192/200 remained within ± 5% of the original value, with *n* = 120/200 within ± 1% (Figures 4I–L). For two *Streptococci* isolates, the N50 value increased from ∼5 to 7.6 kb (a 35% increase) and 12.7 kb (a 60% increase). For *n* = 5/200 isolates, the decrease in the N50 value between 5 and 26% was observed while an increase in the number of reads for all was > 10%.

By far the biggest difference was obtained for the read quality score (Q-score) whereby an increase was observed for the majority (*n* = 185/200, Figures 4E–H) from a mean of 10.22 to 13.12. For *n* = 184/200, the mean read quality increased by > 18%. For the remaining *n* = 16/200 isolates, the original sequencing was performed in June 2016 and basecalled with Guppy v4.5.4 and were all within ± 1%. The highest quality re-basecalled reads included all isolates extracted using the chloroform precipitation method (Figure 4F and Supplementary Table 2) increasing from ∼10.5 to 14, whereas similar ranges were observed for the two-kit based chemical and mechanical extraction methods. Similar increases of mean read quality were observed across the range of GC content and gDNA input (Figures 4K,L).

### Does Re-basecalling Old Oxford Nanopore Technology FAST5 Improve Hybrid *de novo* Assembly?

To determine whether improvements to Unicycler assemblies were observed using the re-basecalled fastq, *n* = 62/200 isolates (across the GC content and ARG range) were selected for additional hybrid *de novo* assembly (Figure 2). Isolates had a range of LR (3,664–1,038,564) and N50 values (176–18,370 bp). The mean read quality for the LR input (*n* = 62/200) increased from 10.33 to 13.01.

In total, *n* = 37/62 of the original basecalled hybrid *de novo* assemblies were marked as a complete chromosome whereas this number increased to *n* = 41/62 with re-basecalled data (Table 2). Circularization of the chromosome improved for two *E. coli* (MIN-073, MIN-076) one *K. pneumoniae* (MIN-105), one *A. baumannii* (MIN-015), and one *S. marcescens* (MIN-165). For *S. marcescens* isolate MIN-164, the initially complete chromosome was not circularized following re-analysis; however, the N50 value remained similar and yielded an improvement in the assembly (circularization) of the plasmid containing *bla*_*NDM*–1_.

**TABLE 2:** The number of isolates per genera selected for hybrid assembly using Unicycler (v0.4.7 and v0.4.9).

Isolates		Complete chromosome	
Species	*n* =	Original hybrid	New hybrid
*Acinetobacter*	7	4	5
*Burkholderia*	2	2	2
*Enterobacter*	4	3	3
*Escherichia*	8	4	6
*Klebsiella*	13	8	9
*Morganella*	1	0	0
*Mycoplasma*	2	1	1
*Pseudomonas*	5	3	3
*Salmonella*	3	2	2
*Serratia*	3	2	2
*Staphylococci*	5	2	2
*Streptococci*	6	3	3
*Ureaplasma*	3	3	3
Total	62	37	41

*The original hybrid assembly uses the original basecalled fastq and the new hybrid genome was generated using Guppy v5.0.11 basecalled reads (from the same fast5 data).*

The assembly N50 metric for *n* = 60/62 repeat hybrid assemblies were similar (within ± 1%) and for *n* = 2/62 isolates (*S. aureus* MIN-170 and *K. pneumoniae* MIN-098) decreased by more than 10%. With the five isolates with > 20 contigs, re-assembly did not improve the number of contigs and for *K. pneumoniae* (MIN-098), the N50 reduced from 3,571,081 to 1,503,698 bp, producing a *de novo* assembly with 30 contigs (compared with the original 29).

There was one isolate with a difference in the number of circularized plasmids (*S. marcescens*, MIN-163). For this isolate, the original *de novo* assembly produced a contigs file with three contigs (1 length = 5,098,586 depth = 1.00x circular = true, 2 length = 110,795 depth = 0.43x circular = true, 3 length = 8,496 depth = 0.50x circular = true), whereas the re-analysis produced two complete contigs (1 length = 5,098,352 depth = 1.00x circular = true, 2 length = 110,797 depth = 0.49x circular = true). No differences were observed in the detection and location of ARG.

We have shown that re-basecalling old MinION data can vastly improve the mean read quality, which can in turn improve the *de novo* assembly. From the *n* = 62 isolate cohort selected for re-hybrid assembly, we have compared four LR assemblers using the basecalled data from Guppy v5.0.11 for *n* = 25/62 isolates. This analysis was performed to determine whether the most accurate ONT reads available (at the time of writing) against the in-house “reference” assembly for each isolate (the *de novo* hybrid assembly from Unicycler v0.4.9, which also used the input LR from Guppy v5.0.11) allows for accurate genome assembly and ARG contextual determination.

### Long Read *de novo* Assemblies

Sixteen LR assembly variations were performed (as outlined in Supplementary Figure 1). SR assemblies were produced for comparative purposes and Supplementary Table 3 summarizes the Quast metrics. Raven performed the quickest and was the least computationally intensive, whereas Canu assemblies took the longest to produce the *de novo* assembly. Quast metrics for the LR assemblies are shown in detail in Supplementary Table 4 and summarized for each assembler in Table 3. The mean sequencing coverage (based on available mapped LR data during Racon polishing within Canu assembly ranged between 9X and 383X (Supplementary Table 5). There were insufficient reads for LR assembly for MIN-169 (*S. aureus*) and partial assemblies for MIN-132 (9X coverage, *M. hominis*).

**TABLE 3:** Comparison of Quast metrics number of contigs and N50 value between the hybrid assembly (internal reference) and the four assemblers used.

Isolate information		Hybrid assembly		Canu		Flye		Miniasm		Raven	
Isolate ID	Species	No contigs	N50	No contigs	N50	No contigs	N50	No contigs	N50	No contigs	N50
MIN-005	*Acinetobacter baumannii*	12	3,525,656	75	2,633,785	8	3,964,326	60	2,774,148	12	3,998,627
MIN-010	*Acinetobacter baumannii*	7	3,820,548	116	1,911,802	4	3,819,441	4	3,817,982	4	3,818,776
MIN-012	*Acinetobacter baumannii*	4	3,878,733	25	797,795	5	3,873,279	23	346,264	14	882,722
MIN-021	*Burkholderia cenocepacia*	5	2,953,509	33	3,020,397	4	2,996,827	4	3,006,784	5	2,996,894
MIN-029	*Enterobacter asburiae*	11	3,233,646	23	4,799,594	5	4,785,704	12	4,785,894	6	4,682,746
MIN-030	*Enterobacter hormaechei*	4	4,716,358	37	4,576,092	3	4,714,883	8	4,263,122	3	4,715,059
MIN-036	*Escherichia coli*	6	4,928,993	166	3,944,955	7	4,927,117	3	4,927,923	3	4,926,621
MIN-040	*Escherichia coli*	4	4,514,829	6	4,677,337	2	4,652,244	2	4,652,852	2	4,652,280
MIN-049	*Escherichia coli*	5	4,768,909	40	4,791,838	5	4,769,105	3	4,773,444	5	4,769,200
MIN-073	*Escherichia coli*	12	4,742,845	12	4,754,605	6	4,744,646	7	4,743,179	4	4,698,004
MIN-088	*Raoultella ornithinolytica*	4	5,399,857	79	5,416,040	6	5,398,098	18	4,815,076	5	5,106,679
MIN-106	*Klebsiella pneumoniae*	8	5,300,304	140	5,323,728	6	5,297,916	235	5,298,979	6	5,298,450
MIN-119	*Klebsiella pneumoniae*	11	5,054,824	113	5,124,097	5	5,103,142	113	5,103,754	5	5,103,899
MIN-129	*Klebsiella quasipneumoniae*	20	5,038,265	91	5,283,547	6	5,255,931	5	5,258,770	3	5,258,391
MIN-132	*Mycoplasma hominis*	1	708,710	13	50,339	1	707,499	9	40,375	11	73,132
MIN-137	*Pseudomonas aeruginosa*	2	6,531,433	9	4,840,050	2	6,527,025	5	2,736,100	2	5,810,542
MIN-156	*Pseudomonas putida*	3	5,944,170	6	6,185,656	2	6,167,022	1	6,167,466	1	6,167,000
MIN-160	*Salmonella enterica*	2	4,634,052	20	4,748,104	2	4,726,543	2	4,727,634	3	4,726,892
MIN-164	*Serratia marcescens*	2	5,099,060	18	1,973,127	2	5,096,271	2	5,097,058	2	5,096,789
MIN-169	*Staphylococcus aureus*	2	2,792,307	73	25,754	41	86,444	5	27,339	39	32,652
MIN-176	*Staphylococcus sciuri*	1	2,933,004	11	2,954,979	1	2,932,657	1	2,933,161	1	2,933,014
MIN-182	*Streptococcus agalactiae*	2	2,024,578	14	711,413	1	2,140,986	3	2,059,889	2	2,062,716
MIN-189	*Streptococcus agalactiae*	3	2,185,475	9	2,263,670	1	2,239,484	1	2,238,695	2	2,180,722
MIN-198	*Ureaplasma parvum*	1	726,862	20	717,827	1	726,458	6	726,694	3	726,167
MIN-201	*Ureaplasma urealyticum*	1	816,741	4	834,700	1	816,472	1	816,845	1	816,642

*Data represent an average of the metrics produced from the Filtlong and Nanofilt assemblies.*

**FIGURE 5:**
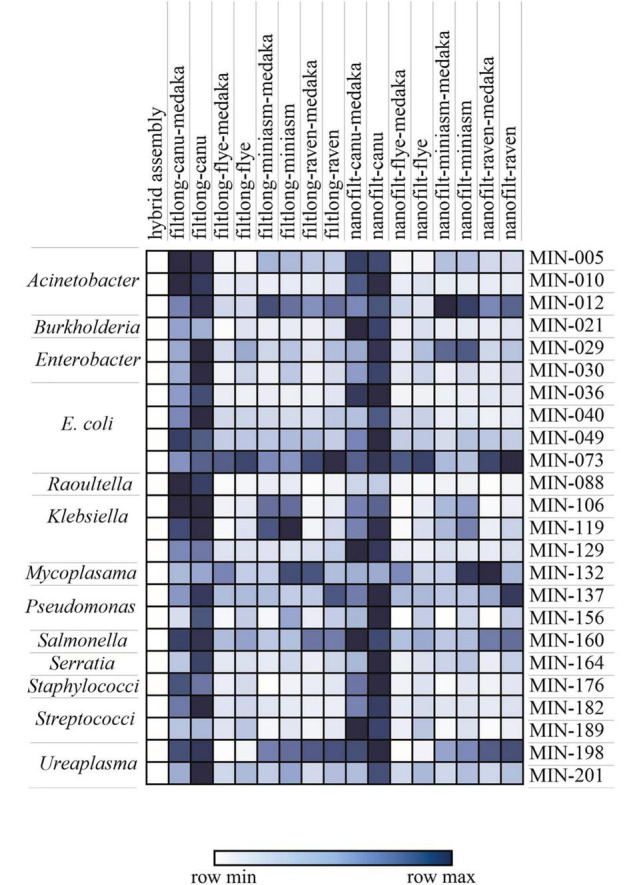
Mash distance matrix comparing the hybrid assembly to each long-read only assembly variation for *n* = 23 isolates (MIN-, only isolates where all assemblies were generated were included). The darker (blue) represents a greater genomic distance. The species/genera are indicated on the left.

Canu produced the most fragmented assemblies with the number of contigs ranging from 2 to 209 (median *n* = 20, mean *n* = 43). Assemblies with the input reads pre-processed from Nanofilt (*n* = 20/25) often generated more fragmented assemblies; however, the N50 values were similar, and in some cases improved with the additional filtering (Supplementary Table 4). Three Canu assemblies failed to complete with the filtlong reads as input; however, assemblies were generated with the Nanofilt reads. Compared with the hybrid *de novo* assembly metrics (Quast), Flye produced the most similar assemblies (Tables 3, 4). Mash genome distances using MinHash (Figure 5) indicate that Canu assemblies least resembled the comparative hybrid assembly (0.002149–0.002730) followed by Miniasm (0.002102–0.002278) and Raven (0.000747–0.001557). On average, Flye assemblies produced the smallest genomic distance (0.000769–0.000930). Of the 59 LR assemblies with < 100 single nucleotide variations (SNVs; against corresponding Unicycler genome), 26 were Flye based, 21 were Raven based, and 12 were Miniasm based (Table 4 and Supplementary Table 6). Conversely, 103 assemblies with > 2,000 SNVs were and 57/103 were Canu-based, with 23 Miniasm-based, 14 Raven-based, and 9 Flye-based (Table 4). The following data provide examples of ARG comparison from assembly variations per isolate.

**TABLE 4:** The mean number of single nucleotide variant (SNVs), insertions and deletions detected from whole genome alignments for each LR assembly against the appropriate Unicycler reference.

Assembly	SNVs	Insertions	Deletions	Total
Filtlong-Canu-Racon	3,334	665	1,878	5,877
Filtlong-Canu-Racon-medaka	4,965	1,340	1,284	7,589
Filtlong-Flye	565	215	987	1,767
Filtlong-Flye-medaka	525	536	125	1,186
Filtlong-Miniasm	3,322	903	2,106	6,331
Filtlong-Miniasm-medaka	3,744	1,114	1,316	6,174
Filtlong-Raven	654	229	973	1,856
Filtlong-Raven-medaka	556	513	145	1,214
Nanofilt-Canu-Racon	3,805	815	2,185	6,805
Nanofilt-Canu-Racon-medaka	6,016	1,585	1,721	9,322
Nanofilt-Flye	579	206	931	1,716
Nanofilt-Flye-medaka	492	481	142	1,115
Nanofilt-Miniasm	2,627	647	1,780	5,054
Nanofilt-Miniasm-medaka	2,881	999	1,100	4,980
Nanofilt-Raven	739	232	995	1,966
Nanofilt-Raven-medaka	669	552	186	1,407

*Data for n = 23 isolates representing isolates where Canu failed were not included in this analysis. Supplementary Table 6 summarizes individual isolate data per assembler.*

### *K. pneumoniae* Long Read Assemblies and Antimicrobial Resistance Genes

For two *K. pneumoniae* isolates (MIN-106 and MIN-119), both Canu and Miniasm assemblers produced contigs files with > 100 contigs. There were > 1 carbapenemase ARG copy detected for both *K. pneumoniae* isolates, among several other ARG. Both Flye and Raven produced genomes with between 5 and 7 contigs (hybrid genomes for these isolates had 8 and 11 contigs).

**FIGURE 6:**
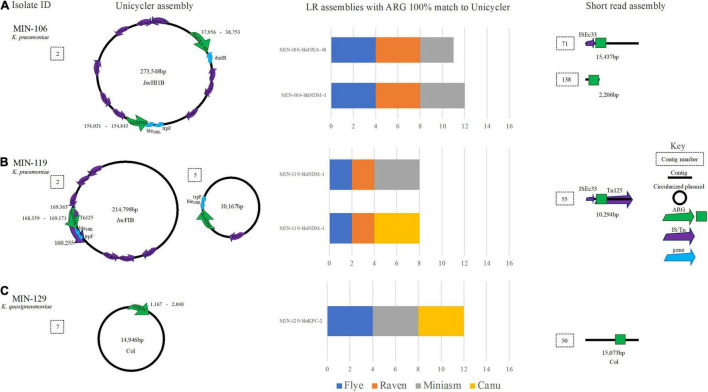
A schematic to compare *Klebsiella* ARG detection from the Unicycler (hybrid) assembly (left), with the short read (SR) assembly (right). The stacked bar graphs (center) denote the number of LR assemblies with a 100% match to the Unicycler assembly. **(A)** MIN-106 with bla_*NDM*−5_, bla_*OXA*−48_ like on the same plasmid, **(B)** MIN-119 with two plasmid both containing bla_*NDM*−1_ and **(C)** MIN-129, a K. quaispneumoniae isolate containing bla_*KPC*−2_.

Two genomes for MIN-106 (Nanofilt, Canu, and Minisam) had a single SNV within the *bla*_*NDM*–1_ gene, whereas *bla*_*OXA*–48_-like, also detected on the same 273,548-bp *Inc*HI1B plasmid (Figure 6A), was successfully identified except for a single SNV in the filtlong Miniasm genome. *bla*_*NDM*–1_ was located upstream of genes *ble* and *trpF* and *bla*_*OXA*–48_-like was located next to a *dmlR* gene (Figure 6A). There were 9 IS elements detected on the 273,548-bp *Inc*HI1B plasmid with ISEc33 located upstream *bla*_*NDM*_ (Figure 6A). The ST was correctly detected when medaka was performed, except for Nanofilt Canu (Filtlong Canu failed to assemble).

For MIN-119, where two copies of *bla*_*NDM*–1_ were identified from the hybrid assembly; one a 214,798-bp *Inc*FIB plasmid with the *bla*_*NDM*–1_ located within a composite transposon Tn125 (also determined from SR only analysis) and second a 10,167-bp plasmid with ISEc33 located upstream of *bla*_*NDM*_ (Figure 6B). None of the LR genomes had > 1 hit for *bla*_*NDM*–1_, and all hits were located on the > 200-kbp *Inc*FIB plasmid. The ST was only correctly assigned when medaka polishing was applied, irrespective of the sequence read filtering tool and assembler combination.

MIN-129, a *K. quasipneumoniae* isolate, had *bla*_*KPC*–2_ gene isolated on a small (14,946 bp) Col-like plasmid (Figure 6C) with > 70% similarity with Col MGD2, a finding also reported previously (Cerdeira et al., 2019). The plasmid was circularized following a hybrid *de novo* assembly (Figure 6C). The plasmid type (Col) was detectable from SR only analysis as the ARG was located on a 15,073-bp contig, similar in length to the Unicycler assembly (Figure 6C). Results were diverse when analyzing the LR assembly variations. *Bla*_*KPC*–2_ was not identified in any of the four combinations (filtering-assembling-polishing) using Raven, with between 2 and 4 contigs generated whereas for the Miniasm and Flye assemblies there were 5–6 contigs. Canu had > 70 contigs and 74 hits (> 90% cov.) for 7 *bla*_*KPC*_ variants across multiple contigs. Of the eight ARG identified on the chromosome from the LR assemblies, seven were identified at 100% cov. (from all assembly variations) including *aac(6′)-lb-cr*. *qnrB1* was identified on the chromosome; however, when screening LR variations, there were between 3 and 4 copies identified. From the hybrid reference, *bla*_*SHV*–12_ was identified on a small linear sequence (4,492 bp), but was only identified from half of the LR assemblies (Canu and Miniasm). Flye assemblies identified a partial match (<30% cov.) to *bla*_*SHV*–187_.

### *Escherichia coli* Long Read Assemblies and Antimicrobial Resistance Genes

**FIGURE 7:**
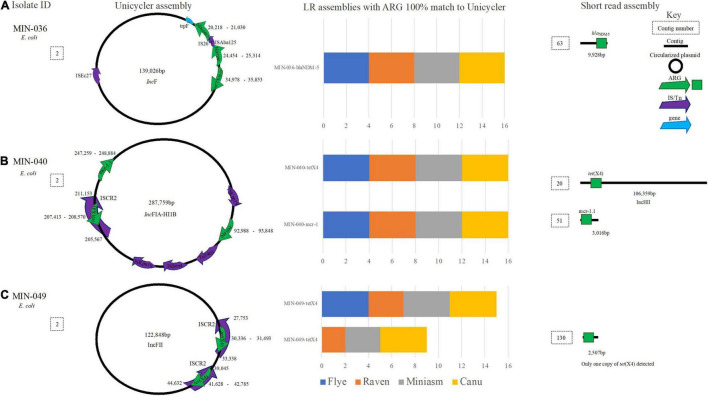
A schematic to compare *E. coli* ARG detection from the Unicycler (hybrid) assembly (left), with the short read (SR) assembly (right). The stacked bar graphs (center) denote the number of LR assemblies with a 100% match to the Unicycler assembly, **(A)** MIN-036 with bla_*NDM*−5_, bla_*TEM*_, and bla_*CTX*−*M*−15_on the same plasmid, **(B)** MIN-040 with tet(X4),mcr-1.1 and bla_*TEM*_ on the same plasmid, **(C)** MIN-049 a plasmid with two copies of tet(X4).

*E. coli* isolate MIN-036 contained *bla*_*NDM*–5_, *bla*_*TEM*_, and *bla*_*CTX*–*M*–15_ on a 139,026-bp plasmid (Figure 7A). All 16 LR assemblies were able to detect all three β-lactamase ARG on the same contig whereas the SR assembly was more fragmented, and *bla*_*NDM*–5_ was detected on a small contig (9,928 bp, Figure 7A). For MIN-040, *tet*(X4) within an ISCR2 element, *bla*_*TEM*_, and mcr-1.1 were detected on a large 287,759-bp *Inc*FIA-HI1B plasmid (Figure 7B). All 16 LR assemblies were able to detect the three ARG on the same contig, whereas the SR assembly was more fragmented (Figure 7B).

Unicycler assembly produced five contigs for MIN-049, with a complete chromosome and three circularized plasmids. Miniasm assemblies produced three contigs, Flye and Raven produced 4–5, and Canu assemblies 35–45 contigs. Two copies of *tet*(X4) were identified through the hybrid assembly, and these were both located on a 122,848-bp *IncFII* plasmid (Figure 7C). From the 16 LR assemblies, there were 26 hits for *tet*(X4). Miniasm and Canu assemblies detected all copies of *tet*(X4), whereas 6/8 were detected for Raven (when Filtlong used), and only a single copy of the ARG was detected in the Flye assemblies (Figure 7C).

MIN-073 had the largest genomic difference between the hybrid *de novo* assembly and the LR assemblies. In nine LR genomes, *bla*_*NDM*–5_ was detected but was absent from the hybrid genome. This finding was unexpected and repeat sequencing across all platforms with single colony gDNA extracted simultaneously would be needed to identify the extent of differences.

### Other Gram-Negative Bacteria Species Long Read Assemblies and Antimicrobial Resistance Genes

**FIGURE 8:**
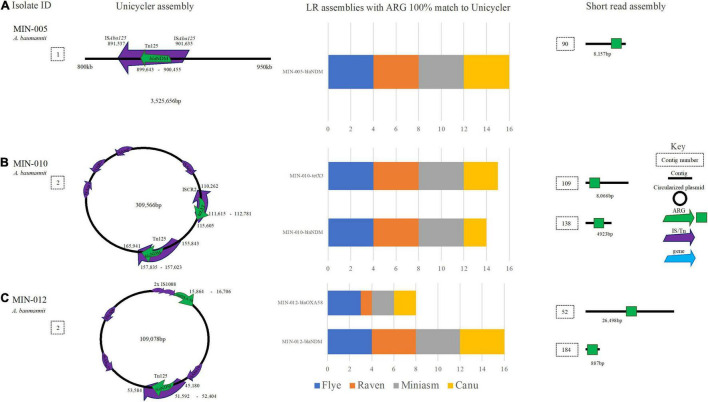
A schematic to compare *A baumannii* ARG detection from the Unicycler (hybrid) assembly (left), with the short read (SR) assembly (right). The stacked bar graphs (center) denote the number of LR assemblies with a 100% match to the Unicycler assembly **(A)** MIN-005 with *bla*NDM located on the chromosome, **(B)** MJN-010 with bla_*NDM*−2_ and tet(X3) located on the same plasmid. **(C)** MIN-012 with bla_*NDM*−1_ and bla_*OXA*−58_ on the same plasmid.

MIN-005 (*A. baumannii*) contained *bla*_*NDM*–1_ within an IS*Aba125*-mediated composite transposon (Tn125) located on the chromosome. Although the ARG (*bla*_*NDM*–1_) was identified at 100% ID and 100% coverage across all 16 LR assemblies, Tn125 was split across at least 2 contigs for all LR assemblies therefore complicating assessment of the genetic context (Figure 8A). Similarly, *A. baumannii* MIN-010 carried *bla*_*NDM*–1_ located in a Tn125 transposon in addition to *tet*(X3) within an ISCR2 element (among several other ARG) on the same 309,566 bp plasmid (Figure 8B). For filtlong and Nanofilt Canu assemblies, a SNV in *bla*_*NDM*–1_ (99.75% ID and cov.) was resolved to 100% ID post medaka. All other assembly variations yielded a 100% match to *bla*_*NDM*–1_. *tet*(X3) was identified in all 16 assemblies at 100% cov. and ID except for in the Filtlong-Miniasm assembly where the gene coverage was 99.91% (100% post medaka). Although it was possible to ascertain the target ARG from MIN-010, the Canu-based assemblies were highly fragmented with a much larger genome size expected for an *A. baumannii* (∼4.7–5 Mb compared with ∼4.2 Mb). ST132 (Pasteur) was correctly assigned for all assemblies. *bla*_*NDM*–1_ located within a Tn125 transposon and upstream IS*Aba16*, and *bla*_*OXA*–58_ located upstream of two IS1008 elements were detected on a 109,078-bp plasmid in MIN-012 (*A. baumannii*) (Figure 8C).

**FIGURE 9:**
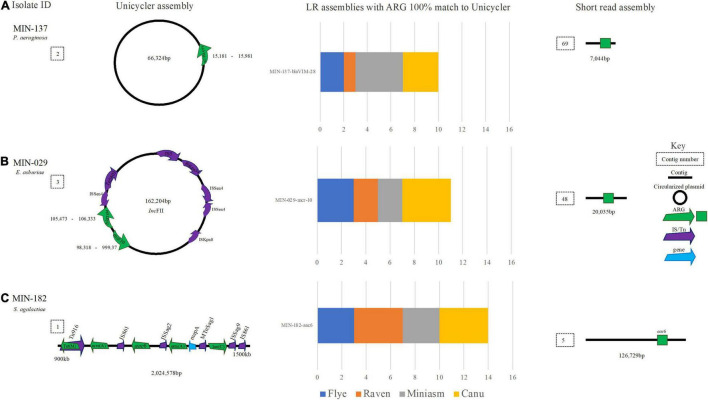
A schematic to compare ARG detection from the Unicycler (hybrid) assembly (left), with the short read (SR) assembly (right). The stacked bar graphs (center) denote the number of LR assemblies with a 100% match to the Unicycler assembly. **(A)** MIN-137, an *P. aemginosa* isolate with *bla*VIM-28 located on a plasmid. **(B)** An *E. asburiae* isolate MIN-029 with blaTEM and mcr-10 located on a plasmid, **(C)** MIN-182 and *S. agalactiae* isolate with aminoglycoside and macrolide antibiotic resistance genes.

A *Pseudomonas aeruginosa* isolate with a *bla*_*VIM*–28_ (MIN-137) assembled (hybrid) into two contigs with a complete chromosome (N50 6,531,433 bp), *bla*_*VIM*–28_ was located on a 66,324 bp plasmid (Figure 9A). The LR assembly data reveal 13/16 *de novo* variations detected *bla*_*VIM*–28_. All Flye, Miniasm, and Canu (when medaka was applied) assemblies detected *bla*_*VIM*–28_ at 100% cov. and > 99.63% ID (indicating some discrepancy in basecalling data), whereas the ARG was only detected from the Filtlong and Raven combination (with and without medaka polishing; Figure 9A).

MIN-029, an *Enterobacter asburiae* isolate with *bla*_*ACT*–2_, *bla*_*TEM*–1_, *fosA*, *OqxA*, and *mcr-10*, was assembled (hybrid) into 11 contigs and the chromosome was not complete (N50 3,233,646 bp). Raven and Flye produced *de novo* assemblies between 5 and 6 contigs, whereas Miniasm and Canu produced assemblies between 5 and 34 contigs. *mcr-10*, *fosA bla*_*ACT*–2_, *bla*_*TEM*–1_, and *OqxA* were identified across all LR assemblies. mcr-10 was identified on a 162,204-bp *Inc*FII plasmid with *bla*_*TEM*_ (Figure 9B).

Although no ARG were detected in MIN-021 (*Burkholderia cenocepacia*), comparison of the LR assemblies revealed that all *de novo* assemblies except Canu-based assemblies produced 4–5 contigs, with the greatest number of SNVs (2,305–14,568) also identified from Canu-based assemblies (compared with 24–894). Unicycler produced five contigs, two of the three chromosomes were complete (3.43, 2.95, and 1.02 Mb), and two were plasmids (136,256 and 41,814 bp). All LR assemblies including Canu were able to assemble the three chromosome contigs of similar lengths to the hybrid genome with N50 values between 2.996 and 3.024 Mb (hybrid N50 2.953 Mb).

### Streptococci Long Read Assemblies and Antimicrobial Resistance Genes

*Streptococcus agalactiae* (MIN-182) assembled (hybrid) into three contigs with an N50 of 2,185,475 bp. Flye and Miniasm assemblies produced a single contig, whereas Raven produced between 1 and 3 contigs. Canu assemblies were more fragmented, between 8 and 9 contigs in length, with a similar N50, but a slightly higher total genome size. Aminoglycoside, macrolide, and tetracycline ARG were detected [*ant(6)-Ia, aph(3′)-III, mre(A), erm(B)*, and *tet(S)]* (Figure 9C). All ARG were detected in the 16 LR assembly variations; however, there were 18 hits for each ARG in total. Duplicate copies identified were detected in the Nanofilt Canu assemblies within different positions of the chromosome.

### Mollicutes Long Read Assemblies and Point Mutations

**FIGURE 10:**
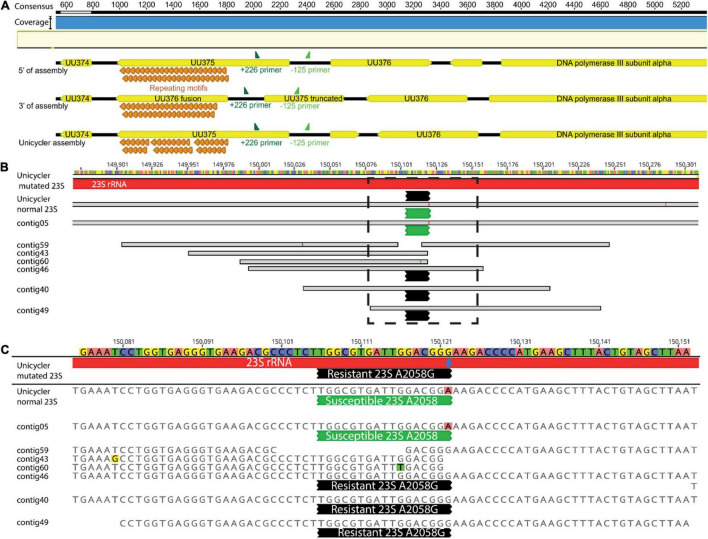
A schematic to visualize **(A)** 3′ and 5′ end s of the Canu-based assemblies indicating they have both, versions of the UU375 rearrangement vs. Unicycler (as well as the adjacent non-inverted genes) at the beginning and end of the assembly, PAGKEQ repeat motifs (orange) and primers +226 and –125 (dark and light green, respectively) are shown for context of the small inverted region; **(B)** the alignment short reads to the Unicycler assembly of MIN-201 (Ureaplasma urealyticum), bases representing mismatches in base calls are identified with different color highlighting and contigs containing 23S rRNA mutations at A2058G (blue arrow) mediating resistance are identified at the end of the black probed region, while contigs containing wild type susceptible 23S rRNA A2058 are identified by green probed regions, **(C)** shows a higher resolution of the region identified by a dotted box in **(B)**.

Progressive Mauve within Geneious was performed to examine LR sequence identity and gene annotation for Mollicutes. LR data for *U. parvum* (MIN-198) identified inversion events adjacent to the UU375 gene locus, a known phenomenon whereby *Ureaplasma* spp. undergo genomic inversion events to escape immune response (Zimmerman et al., 2009). The orientation in the Unicycler assembly (the only one influenced by short-read data) was opposite to Flye-based assemblies. Canu-based assemblies were, however, found to have both versions of the UU375 inversion (as well as the adjacent non-inverted genes) at the beginning and end of the assembly resulting in an additional 8-kb sequence length (Figure 10A).

By comparison, five assemblies (Flye and Unicycler) for *U. parvum* (MIN-198) (286,731 LR) were single contigs within 546 bp shorter to 265 bp longer in total length with an identity of 99.9% (relative to Unicycler). The UU375 gene also has the unusual characteristic of containing variable numbers of PAGKEQ repeats at the C terminus and assemblies using Filtlong were nearly identical to Unicycler (39 or 40 repeats) relative to assemblies that used Nanofilt that gave 54 repeats. However, direct annotation using 100% identity relative to the Unicycler-assembled genome gave a range of 52.6–77.1% of the CDS for annotation. The predominant error failure to annotate was insertion or omission of an extra base leading to frame-shift for the ORF. This phenomenon was also observed for the *U. urealyticum* genome (MIN-201; 106,356 LR), where 12 assemblies (all except those using Canu) were single full-genome assemblies between 283 bp shorter and 186 bp longer (relative to Unicycler) with an overall identity of 99.4%. However, this relates to a much lower annotation identity of 34.7–49.4% CDS relative to Unicycler. Again, the predominant error was random insertion or omission of a single base within the ORF. All assemblies were able, however, to pick out a macrolide resistance–mediating mutation for MIN-201: all showed one 23S rRNA carrying the A2058G resistance mutation and one 23S rRNA with a susceptible sequence. Base insertion/omission, however, meant that 100% identity for the entire 23S rRNA gene was never observed for any of the LR assemblies (Figures 10B,C).

Both *Ureaplasma* spp. genomes represent the lowest GC% (25.4–25.7) and the annotation test for MIN-189 (*Streptococcus agalactiae*; 209,392 LR) that has a GC content of 35.9% was much better for annotation identity giving 68.5–89.3% CDS annotation (overall identity of assemblies 98%), although Unicycler assembly generated 2 contigs (2,024,578 and 117,550 bp) while Flye-based contigs generated single full genome assemblies. Failure to achieve 100% annotation was again caused by insertion or omission of single bases within ORFs. The accuracy of base calling appears to be GC content sensitive as the *K. pneumoniae* isolate (MIN-119; 96,163 LR available; GC content 57.1%) gave a range of rate of 100% identical annotation relative to Unicycler of 57.3–93.3%, despite having fewer LR than MIN-198, MIN-201, and MIN-189. The highest conservation of ORF integrity relative to Unicycler was obtained where medaka was incorporated.

### The 96 Rapid Barcoding Kit: R9 vs. R10 for Output Yield and Sequence Read Quality

With the release of the ONT 96 rapid kit allowing for a larger multiplex, we performed a comparison of the same sequencing library on both R9 and R10 flow cells. In total, *n* = 53 isolates of *K. pneumoniae, E. cloacae*, and *E. coli* were selected (gDNA range between 12.4 and > 100 ng/μl) initially pooled to produce a concentrate of 423 ng/μl (total 4,653 ng) and diluted to produce six aliquots of the same library loading ∼775 ng of DNA per flow cell. In parallel, we loaded the same sequencing library (with R10 flow cells) on a standard laptop computer and the Mk1C, therefore loading three flow cells in total. The R9 flow cell (with 1,440 active pores) produced the greatest yield of data with 173.7 GB fast5 (as cited previously, Xu et al., 2021), with R10 producing 64.3 GB (1,322 pores) and 61.8 GB (922 pores). After performing the flow cell washes, we loaded the same sequencing library to all three flow cells and re-ran MinKNOW with the same parameters producing an additional 10.3 GB on R9 (67 pores) and on R10 2.4 GB (325 pores) and 5.2 GB (183 pores) fast5 data.

The percentage of reads > Q12 was greatest for data recovered from the R9.4 flow cell at 72%, whereas the two R10 flow cells (laptop and Mk1C) were 60 and 61%, respectively (Table 5 and Supplementary Table 7). The mean number of reads generated on the R9.4 flow cell was > 37 K, whereas the R10 flow cells generated between 8.8 and 12.3 K reads. The N50 values observed were larger than all previous MinION data at 15,422 (merged average, Table 5). The highest individual N50 was recovered from the R9.4 flow cell at 22,580 bp (for the R10 laptop the N50 was similar at 22,263 and 20,910 bp using the Mk1C). Four isolates had an N50 < 10 kb (but greater than 7.5 kb) and these were among the isolates with a gDNA input below the recommended input (between 12.4 and 20 ng/μl).

**TABLE 5:** Sequence read metrics comparing the recovery yield, mean read quality (Q score), and N50 averages for *n* = 53 GNB isolates sequenced on three different flow cells, and instruments.

Flow cell/computer	Read metric	Mean data per flow cell
FAQ06625 (Laptop), R10	Number of reads	12,308
	N50 (bp)	15,218
	Mean read length (bp)	7,373
	Mean read quality	12
	% > Q12	60%
FAQ06664 (Mk1C), R10	Number of reads	8,889
	N50 (bp)	14,850
	Mean read length (bp)	7,165
	Mean read quality	12
	% > Q12	61%
FAQ23227 (PC), R9.4	Number of reads	37,270
	N50 (bp)	15,629
	Mean read length (bp)	7,188
	Mean read quality	13
	% > Q12	72%
Merged data	Number of reads	58,466
	N50 (bp)	15,422
	Mean read length (bp)	7,222
	Mean read quality	13
	% > Q12	68%

### Oxford Nanopore MinION Sequencing: A Cost Analysis

Experience working in LMICs has shown that non-profit LMIC laboratories can most reliably be supplied by purchasing consumables *vi*a our (United Kingdom) laboratories, as LMIC supply chains often yield logistical challenges that mainstream scientific suppliers are averse to. Compounding this, we have also found that suppliers in many LMICs are resellers, which charge very large premiums. For these reasons, our general practice, and resulting pricing, is for procurements *via* purchasing frameworks accessible to United Kingdom universities and not-for-profit institutions. The total costs generated this way can be seen in Table 6.

**TABLE 6:**
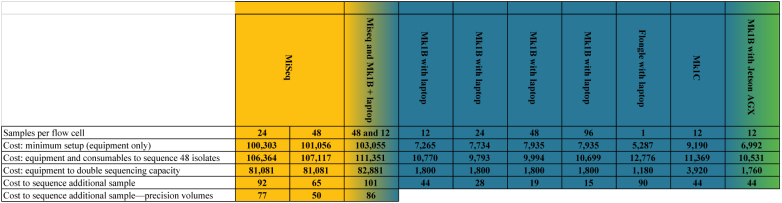
A cost analysis of MiSeq, Mk1B, and Mk1C sequencing using various flow-cell loads.

*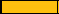 = Illumina Technology; 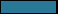 = Oxford Nanopore Technology; 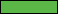 = Nvidia Technology All pricing in GBP (£) as of August 2021. The precision protocol entails halving reagent volumes from the Nextera library preparation kit’s manufacturer-provided SOP and reducing SPRI beads from 22.5 to 11.5 μl/sample. Among 6 suppliers and 17 models of filter tips covering various ranges within 0.5–200 μl, only 4 models (Appleton Woods ACA659, ACT659, ACT619, and Starlabs S1120-3810) yielded DNA concentrations sufficiently conformant to carry on to sequencing with this method (data not shown). Our groups have not yet attempted to create a similarly reagent-optimized ONT protocol.*

The MiSeq was an exception to UK pricing, for two primary reasons: (1) MiSeqs are far too sensitive and valuable to transport with economy shipping partners and (2) for LMIC laboratories we have collaborated with, Illumina strongly recommended using local Illumina Service Partners, which provide delivery, siting, and commissioning of the instrument; Illumina could not otherwise guarantee safe arrival or installation. Climate control requirements for the MiSeq were also much more stringent than a Nanopore system, including temperature, humidity, and air quality requirements. Our research team expects £5,000–9,000 in additional subcontracting costs to meet these minimum requirements. Given our focus on minimizing costs for LMICs, we used the costs to independently procure a suitable air conditioner (RS Components 1875320, £701) and dehumidifier (RS Components 1245210, £258). Housing a MiSeq in a laboratory containing HEPA- or ULPA-filtered laminar flow hoods will provide background air filtration, allowing a dehumidifier and air conditioner to meet MiSeq’s environmental operating requirements of maintaining a 22 ± 3°C ambient temperature, a 30–75% non-condensing relative humidity, and an IEC pollution degree of ≤ 2. ONT provides a single environmental condition for the Mk1B sequencer: a temperature range of 18–24°C.

## Discussion

Herein, we have shown that despite clear improvement with ONT sequencing and basecalling, hybrid assemblies are still essential to whole-genome AMR research. Due to high associated costs (Table 6) and/or instruments access limitations, unfortunately, this approach is not feasible for many. LR assemblies can, in many cases, be informative for ARG screening; however, the choice of trimmer, assembler, and polisher considerably influences generated data. There are substantial differences in LR *de novo* assemblies as determined by the genomic distances (Figure 5), also evidenced by Lipworth et al. (2020) and Wick and Holt (2021) *via* read mapping. SNVs and insertions in LR assemblies were predominantly seen in the poly A/T regions, particularly evident for Mollicutes suggesting that basecalling errors (and therefore LR assembly errors) could be exacerbated in bacterial species with a lower GC content.

While ONT recommend an input of ∼50 ng/μl for their rapid kits, we often generated > 100,000 reads with an input yield of 20 ng/μl, and even as low as < 5 ng/μl, particularly for the Mollicutes. For best results, we recommend pooling low yield samples with other isolates > ∼20 ng/μl. ONT’s rapid kits are easier to use compared with the native kit (in terms of reduced third party reagents and equipment) while producing longer reads and, importantly, improve the recovery of small plasmids (Wick et al., 2021b), often harboring ARGs.

Kit-based extractions often generated sufficient high-quality gDNA (Figure 4). Depending on species, additional enzymatic treatments and/or bead beating may be beneficial. The gDNA extraction method does influence the length and number of reads (Figure 3); however, we found that the N50 values largely correlated to genome size (Figure 2). With adjustments to our protocol,^1^ our read length N50 values improved markedly, up to 20 kb.

The most laborious gDNA extraction to perform and reproduce was chloroform precipitation. While we observed the highest N50 values (Figure 4), gDNA was difficult to pipette, we were unable to use SPRI, and we were not always able to produce closed assemblies. From our experience, the use of the Qiagen’s kit-based DNA extraction produces high-quality, large fragment gDNA. Kit-based extractions can also be performed with minimum infrastructure/equipment, needing access only to the supplied extraction kit, a heat block, fridge/freezer, microcentrifuge, Eppendorf tubes, and pipettes.

Often, LRS is performed at a later stage, selected following SR analysis. This introduces the possibility of extracting a different colony following recovery from frozen stocks. We have experienced this in the study (MIN-073) and has been reported elsewhere (Wick et al., 2017; Wick and Holt, 2021); therefore, it is recommended that gDNA is aliquoted upon extraction to allow the same extract to be sequenced for both SR and LR. Furthermore, the use of kits produces DNA that can easily be used for both platforms.

With improvements in LR basecalling (Figure 4), ONT sequencing can be suitable for downstream microbial analyses and AMR surveillance for a wide range of species including Mollicutes. If relying on LRS data, our results support a recommendation to perform Flye and Raven, concordant with other recent findings (Wick and Holt, 2021). Raven was the quickest and easiest tool, the assemblies often produced accurate ARG information, and previous work supports the assembler (Chen et al., 2020); however, smaller plasmids were not always assembled. We noticed this during our analysis, and this has also been reported elsewhere (Wick et al., 2021b). Mixed results were obtained for isolates with multiple copies of the same ARG, and in one example Flye was not able to detect > 1 copy of ARG whereas Raven was. Ideally, and if computing resources are readily available, running Trycycler on various iterations of LR assemblies may further provide a consensus assembly (Wick et al., 2021a). Polishers are recommended to improve the quality of the genome, and during this study we used Racon and medaka, often recommended for ONT reads (Nicholls et al., 2019; Latorre-Pérez et al., 2020). We found ST, ARG, and gene annotation data were greatly improved when medaka was applied emphasizing the importance of including multiple polishing rounds. Nanopolish is another option, but as a polisher reliant on raw FAST5 data, this remains extremely computationally bottlenecked (Hu et al., 2021). There are many recent publications promoting alternative LR polishers, and early data from Homopolish (March 2021) indicate that combining it with preliminary correction of random sequencing errors by Racon or Marginpolish yielded results superior to both Nanopolish and medaka (Huang et al., 2021). These further suggest that ONT sequencing alone will soon be sufficient for downstream microbiological and epidemiological studies.

Bioinformatics analysis remains the primary bottleneck for many users. The Mk1C combines on-board compute and offline mode, beneficial in areas with intermittent internet restrictions/limitations. Combining Mk1B and Nvidia Jetson platforms yields more computational throughput at a lower cost, but is less streamlined (Benton, 2021). If broadband internet is readily accessible, but computing resources are not available and genome assembly not desirable, ONT offers downstream ARG content analyses *via* the EPI2ME WIMP platform.

Using ONT’s 12-index rapid kit, high-quality hybrid assemblies can be generated using a fraction of the total sequencing available within a flow cell (Lipworth et al., 2020). We build upon this work using the newer 96-index rapid-kit on R9.4 and R10.3 simultaneously, supporting the use of heterogeneous barcodes before and after washing ONT flow cells to further reduce cost and minimize read contamination.

While the benefits of nanopore sequencing for hybrid assemblies are recognized, developing high-quality, nanopore-only assemblies will be of particular benefit to LMICs due to cost. While our laboratories have no direct experience with the flongle, a small flow cell with roughly 1/10 the channels of a standard R9.4.1 MinION flow cell, equipment to perform singleplexing with one can be purchased for ∼£5,000. A broadly more useful nanopore setup would require ∼£11,000 (enough consumables to multiplex 48 isolates at 12 isolates per flow cell and access to fluorometry-based DNA QC) as compared with ∼£110,000 on a MiSeq (extensive installation process, additional pieces of costly equipment, and reagents for 48 samples on a 600-cycle flow cell) (Table 6). The latter is outside most LMIC laboratory budgets.

Another benefit of ONT in LMICs is its lack of environmental constraints. ONT provides a temperature range of 18–24°C for Mk1B operation, caveated with “Users can adapt this for other temperature requirements” such as being used in Antarctic in the open (Huang et al., 2021). Successful use in microgravity on the International Space Station (Castro-Wallace et al., 2017; Stahl-Rommel et al., 2021) similarly bolsters this adaptability.

This work highlights the influence of gDNA, quality/length of reads, and choice of LR assemblers in ARG/AMR data. This study focused on drawing comparisons between various LR assemblies, the corresponding hybrid (Illumina MiSeq and ONT) assembly and, where appropriate, the SR assembly, with particular focus on the presence/absence of targeted ARG (Figures 6–10). Limitations of this study include a non-equal sample size for the number of different species and gDNA extraction method, a focus on carbapenemase GNB and/or *K. pneumoniae* and *E. coli* as per the research group’s interest, and a wide variety of read coverage/number and length of LR. Future work is necessary to determine the minimal sequencing depth to determine ARGs across the LR assembly variations.

## Conclusion

Our earliest intention in undertaking this work was to determine LRS’s place in current and future AMR genomics with a focus applicable, but not wholly restricted, to LMIC collaborators. A strong background collaborating with LMIC laboratories allowed us to quickly narrow our view to ONT’s offerings due to cost and practicality advantages. Two chemistries are currently available for ONT flow cells, R10.3 and R9.4.1. The R10.3 has shown promise for homologous regions in some publications, and the newly released R10.4 has shown model accuracy above 99%, but the well-proven yield gains of R9.4.1 chemistry combined with rapidly improving accuracy due to an influx of filtering, basecalling, and polishing breakthroughs leads us to tentatively recommend R9.4.1 until R10 chemistry has undergone similarly significant refinement—both *in vitro* and *in silico*.

Of the filtering, basecalling, and polishing tested, we recommend avoiding using Canu or Raven alone when assembling LR to determine the presence of ARG. Overall, Flye is the best assembler we tested for LR assemblies. Guppy basecalling has clearly and consistently improved since its release. As re-basecalling old FAST5 data benefits from these improvements, we wholeheartedly recommend that FAST5 data from ongoing experiments be saved for re-basecalling.

While ongoing refinement of ONT chemistry may allow complete transition to LR *de novo* assembly in the future, hybrid assemblies currently yield tangible Q-score benefits and sometimes drastic consensus of assembly improvements.

## Data Availability Statement

The datasets presented in this study can be found in online repositories. The names of the repository/repositories and accession number(s) can be found below: https://www.ncbi.nlm.nih.gov/, PRJNA769286; https://www.ebi.ac.uk/ena, PRJEB47698.

## Author Contributions

IB and KS designed the study and wrote the manuscript. KS, IB, and OS performed the bioinformatics analysis. KS, EP, and IB performed the microbial culture and whole genome sequencing. EP, OS, and TW guided the analysis and revision to the article. All authors approved the article.

## Conflict of Interest

The authors declare that the research was conducted in the absence of any commercial or financial relationships that could be construed as a potential conflict of interest.

## Publisher’s Note

All claims expressed in this article are solely those of the authors and do not necessarily represent those of their affiliated organizations, or those of the publisher, the editors and the reviewers. Any product that may be evaluated in this article, or claim that may be made by its manufacturer, is not guaranteed or endorsed by the publisher.

## Footnotes

1
dx.doi.org/10.17504/protocols.io.byhnpt5e

## References

[B1] Benton M. (2021). *Nvidia Jetson Nanopore Sequencing: A Place to Collate Notes and Resources of Our Journey Into Porting Nanopore Sequencing Over to Accessible, Portable Technology.* San Francisco, CA: GitHub.

[B2] Bortolaia V. Kaas R. S. Ruppe E. Roberts M. C. Schwarz S. Cattoir V. (2020). ResFinder 4.0 for predictions of phenotypes from genotypes. *J. Antimicrobial Chemotherapy* 75 3491–3500. 10.1093/jac/dkaa34 PMC7662176 32780112

[B3] Cardiff University (2021). *Cardiff University eMarketplace.* Cardiff: Cardiff University.

[B4] Castro-Wallace S. L. Chiu C. Y. John K. K. Stahl S. E. Rubins K. H. McIntyre A. B. R. (2017). Nanopore DNA sequencing and genome assembly on the international space station. *Sci. Rep.* 7:18022. 10.1038/s41598-017-18364-18360 PMC5740133 29269933

[B5] Cerdeira L. T. Lam M. M. C. Wyres K. L. Wick R. R. Judd L. M. Lopes R. (2019). Small IncQ1 and col-like plasmids harboring blaKPC-2 and non-Tn4401 elements (NTEKPC-IId) in high-risk lineages of *Klebsiella pneumoniae* CG258. *Antimicrob Agents Chemother.* 63:e02140-18. 10.1128/AAC.02140-2118 PMC6395902 30602517

[B6] Chan A. P. Choi Y. Clarke T. H. Brinkac L. M. White R. C. Jacobs M. R. (2020). AbGRI4, a novel antibiotic resistance island in multiply antibiotic-resistant *Acinetobacter baumannii* clinicl isolates. *J. Antimicrob Chemother.* 75 2760–2768. 10.1093/jac/dkaa266 32681170 PMC7556812

[B7] Chen Z. Erickson D. L. Meng J. (2020). Benchmarking long-read assemblers for genomic analyses of bacterial pathogens using oxford nanopore sequencing. *Int. J. Mol. Sci.* 21:9161. 10.3390/ijms21239161 33271875 PMC7730629

[B8] Chen Z. Erickson D. L. Meng J. (2021). Polishing the Oxford Nanopore long-read assemblies of bacterial pathogens with Illumina short reads to improve genomic analyses. *Genomics* 113 1366–1377. 10.1016/j.ygeno.2021.03.018 33716184

[B9] DAG Hammarskjöld Foundation (2019). *When the Drugs Dont Work Antibiotic Resistance as a Global Development Problem.* Uppsala, NY: Dag Hammarskjöld Foundation

[B10] De Coster W. DHert S. Schultz D. T. Cruts M. Van Broeckhoven C. (2018). NanoPack: visualizing and processing long-read sequencing data. *Bioinformatics* 34 2666–2669. 10.1093/bioinformatics/bty149 29547981 PMC6061794

[B11] de Jesus J. G. Giovanetti M. Faria N. R. Alcantara L. C. J. (2019). Acute vector-borne viral infection: zika and MinION surveillance. *Microbiol. Spectr.* 7 1–11 10.1128/microbiolspec.ame-0008-2019 31400093 PMC10957199

[B12] Diaconu E. L. Carfora V. Alba P. Di Matteo P. Stravino F. Buccella C. (2020). Novel IncFII plasmid harbouring blaNDM-4 in a carbapenem-resistant *Escherichia coli* of pig origin. Italy. *J. Antimicrob Chemother.* 75:3475. 10.1093/jac/dkaa374 32835381 PMC7662189

[B13] Faccone D. Martino F. Albornoz E. Gomez S. Corso A. Petroni A. (2020). Plasmid carrying mcr-9 from an extensively drug-resistant NDM-1-producing *Klebsiella quasipneumoniae* subsp. quasipneumoniae clinical isolate. *Infect. Genet. Evol.* 81:104273. 10.1016/j.meegid.2020.104273 32145334

[B14] Gurevich A. Saveliev V. Vyahhi N. Tesler G. (2013). QUAST: quality assessment tool for genome assemblies. *Bioinformatics* 29 1072–1075. 10.1093/bioinformatics/btt086 23422339 PMC3624806

[B15] Hu K. Huang N. Zou Y. Liao X. Wang J. (2021). MultiNanopolish: refined grouping method for reducing redundant calculations in Nanopolish. *Bioinformatics* 37 2757–2760. 10.1093/bioinformatics/btab078 33532819

[B16] Huang Y. T. Liu P. Y. Shih P. W. (2021). Homopolish: a method for the removal of systematic errors in nanopore sequencing by homologous polishing. *Genome Biol.* 22:95. 10.1186/s13059-021-02282-2286 PMC8011154 33789731

[B17] IAGC (2019). *No Time to Wait Securing the Future from Drug Resistant Infections.* Houston, TX: IAGC.

[B18] Johansson M. H. K. Bortolaia V. Tansirichaiya S. Aarestrup F. M. Roberts A. P. Petersen T. N. (2020). Detection of mobile genetic elements associated with antibiotic resistance in *Salmonella enterica* using a newly developed web tool: MobileElementFinder. *J. Antimicrobial Chemotherapy* 76 101–109. 10.1093/jac/dkaa390 33009809 PMC7729385

[B19] Juma M. Sankaradoss A. Ndombi R. Mwaura P. Damodar T. Nazir J. (2021). Antimicrobial resistance profiling and phylogenetic analysis of *Neisseria gonorrhoeae* clinical isolates from kenya in a resource-limited setting. *Front. Microbiol.* 12:647565. 10.3389/fmicb.2021.647565 34385981 PMC8353456

[B20] Krueger F. (2018). *Trimgalore.* San Francisco, CA: GitHub.

[B21] Lai C. C. Chen S. Y. Ko W. C. Hsueh P. R. (2021). Increased antimicrobial resistance during the COVID-19 pandemic. *Int. J. Antimicrobial Agents* 57:106324. 10.1016/j.ijantimicag.2021.106324 33746045 PMC7972869

[B22] Latorre-Pérez A. Villalba-Bermell P. Pascual J. Vilanova C. (2020). Assembly methods for nanopore-based metagenomic sequencing: a comparative study. *Sci. Rep.* 10:13588. 10.1038/s41598-020-70491-70493 PMC7423617 32788623

[B23] Li H. Durbin R. (2009). Fast and accurate short read alignment with burrows - wheeler transform. *Bioinformatics* 25 1754–1760. 10.1093/bioinformatics/btp324 19451168 PMC2705234

[B24] Li H. Handsaker B. Wysoker A. Fennell T. Ruan J. Homer N. (2009). The sequence alignment / map format and SAMtools. *Bioinformatics* 25 2078–2079. 10.1093/bioinformatics/btp352 19505943 PMC2723002

[B25] Lin H. N. Hsu W. L. (2020). GSAlign: an efficient sequence alignment tool for intra-species genomes. *BMC Genomics* 21:182. 10.1186/s12864-020-6569-6561 PMC7041101 32093618

[B26] Lipworth S. Pickford H. Sanderson N. Chau K. K. Kavanagh J. Barker L. (2020). Optimized use of Oxford nanopore flowcells for hybrid assemblies. *Microbial Genomics* 6:mgen000453. 10.1099/MGEN.0.000453 33174830 PMC7725331

[B27] Nicholls S. M. Quick J. C. Tang S. Loman N. (2019). Ultra-deep, long-read nanopore sequencing of mock microbial community standards. *GigaScience* 8:giz043. 10.1093/GIGASCIENCE/GIZ043 31089679 PMC6520541

[B28] Okonechnikov K. Conesa A. García-Alcalde F. (2016). Qualimap 2: advanced multi-sample quality control for high-throughput sequencing data. *Bioinformatics* 32 292–294. 10.1093/bioinformatics/btv566 26428292 PMC4708105

[B29] Ondov B. D. Treangen T. J. Melsted P. Mallonee A. B. Bergman N. H. Koren S. (2016). Mash: fast genome and metagenome distance estimation using MinHash. *Genome Biol.* 17:132. 10.1186/s13059-016-0997-x 27323842 PMC4915045

[B30] O’Neill J. (2016). Tackling drug-resistant infections globally final report and recommendations. *Rev. Antimicrobial Reistance* 7:110. 10.4103/2045-080x.186181

[B31] Quick J. Loman N. J. Duraffour S. Simpson J. T. Severi E. Cowley L. (2016). Real-time, portable genome sequencing for Ebola surveillance. *Nature* 530 228–232. 10.1038/nature16996 26840485 PMC4817224

[B32] Sadek M. Nariya H. Shimamoto T. Kayama S. Yu L. Hisatsune J. (2020). First genomic characterization of blavim-1 and mcr-9-coharbouring *Enterobacter hormaechei* isolated from food of animal origin. *Pathogens* 9:687. 10.3390/pathogens9090687 32842587 PMC7558541

[B33] Seemann T. (2014). Prokka: rapid prokaryotic genome annotation. *Bioinformatics* 30 2068–2069. 10.1093/bioinformatics/btu153 24642063

[B34] Seemann T. (2018). *Shovill.* San Francisco, CA: GitHub

[B35] Seemann T. (2019a). *ABRicate.* San Francisco, CA: GitHub.

[B36] Seemann T. (2019b). *mlst, Github.* San Francisco, CA: GitHub.

[B37] Stahl-Rommel S. Jain M. Nguyen H. N. Arnold R. R. Aunon-Chancellor S. M. Sharp G. M. (2021). Real-time culture-independent microbial profiling onboard the international space station using nanopore sequencing. *Genes* 12:106. 10.3390/genes12010106 33467183 PMC7830261

[B38] Tegha G. Ciccone E. J. Krysiak R. Kaphatika J. Chikaonda T. Ndhlovu I. (2021). Genomic epidemiology of *Escherichia coli* isolates from a tertiary referral center in lilongwe. Malawi. *Microb Genom.* 7:mgen000490. 10.1099/mgen.0.000490 33295867 PMC8115906

[B39] Telatin A. Fariselli P. Birolo G. (2021). Seqfu: a suite of utilities for the robust and reproducible manipulation of sequence files. *Bioengineering* 8:59. 10.3390/bioengineering8050059 34066939 PMC8148589

[B40] Vaser R. Sović I. Nagarajan N. Šikić M. (2017). Fast and accurate de novo genome assembly from long uncorrected reads. *Genome Res.* 27 737–746. 10.1101/gr.214270.116 28100585 PMC5411768

[B41] Vasiljevic N. Lim M. Humble E. Seah A. Kratzer A. Morf N. V. (2021). Developmental validation of Oxford nanopore technology MinION sequence data and the NGSpeciesID bioinformatic pipeline for forensic genetic species identification. *Forensic Sci. Int. Genet.* 53:102493. 10.1016/j.fsigen.2021.102493 33770699

[B42] WHO Antimicrobial Resistance Division (2014). *Antimicrobial Resistance Global Report on Surveilance.* Geneva: WHO.

[B43] Wick R. R. (2018a). *Filtlong.* San Francisco, CA: GitHub

[B44] Wick R. R. (2018b). *Porechop 0.2.4*. San Francisco, CA: GitHub.

[B45] Wick R. R. Holt K. E. (2021). Benchmarking of long-read assemblers for prokaryote whole genome sequencing. *F1000Research* 8:2138. 10.12688/f1000research.21782.4 31984131 PMC6966772

[B46] Wick R. R. Schultz M. B. Zobel J. Holt K. E. (2015). Bandage: interactive visualization of de novo genome assemblies. *Bioinformatics* 31 3350–3352. 10.1093/bioinformatics/btv383 26099265 PMC4595904

[B47] Wick R. R. Judd L. M. Gorrie C. L. Holt K. E. (2017). Completing bacterial genome assemblies with multiplex MinION sequencing. *Microbial Genomics* 3:e000132. 10.1099/mgen.0.000132 29177090 PMC5695209

[B48] Wick R. R. Judd L. M. Cerdeira L. T. Hawkey J. Méric G. Verzina B. (2021a). Trycycler: consensus long-read assemblies for bacterial genomes. *Genome Biol.* 22:266. 10.1186/s13059-021-02483-z 34521459 PMC8442456

[B49] Wick R. R. Judd L. M. Wyres L. M. Holt K. E. (2021b). Recovery of small plasmid sequences *via* Oxford Nanopore sequencing. *Microbial Genom.* 7:000631. 10.1099/mgen.0.000631 PMC8549360 34431763

[B50] Xu F. Ge C. Li S. Tang S. Xingwen W. Luo H. (2021). Evaluation of nanopore sequencing technology to identify *Salmonella enterica Choleraesuis var*. *Kunzendorf* and *Orion var*. 15+, 34+. *Int. J. Food Microbiol.* 346:109167. 10.1016/j.ijfoodmicro.2021.109167 33774575

[B51] Yu X. Zhang W. Zhao Z. Ye C. Zhou S. Wu S. (2019). Molecular characterization of carbapenem-resistant *Klebsiella pneumoniae* isolates with focus on antimicrobial resistance. *BMC Genomics* 20:822. 10.1186/s12864-019-6225-9 31699025 PMC6839148

[B52] Zimmerman C. U. R. Stiedl T. Rosengarten R. Spergser J. (2009). Alternate phase variation in expression of two major surface membrane proteins (MBA and UU376) of *Ureaplasma parvum* serovar 3. *FEMS Microbiol. Lett.* 292 187–193. 10.1111/j.1574-6968.2009.01505.x 19220471

