# Supplementary material for: Comparing Long-Read Assemblers to Explore the Potential of a Sustainable Low-Cost, Low-Infrastructure Approach to Sequence Antimicrobial Resistant Bacteria With Oxford Nanopore Sequencing
Source: Front Microbiol. 2022 Mar 3;13:796465. doi: 10.3389/fmicb.2022.796465 (PMC8928191; doi:10.3389/fmicb.2022.796465)
Supplement: Supplementary file 1 [file Data_Sheet_1.zip › Frontiers-SupplementaryMethods.docx]

**Materials and methods**

**Bacterial culture and gDNA extraction**

Bacterial isolates were plated from frozen stocks onto selective and differential agar (using antimicrobial susceptibility data) and incubated overnight at 37°C. Three gDNA extraction methods were performed depending bacterial species and extraction date. Isolates cultured between 2018-2021 were included and pseudoanonymised.

*Chloroform precipitation*

Chloroform precipitation was performed between 2018-2019. Briefly, a 10μl loop of bacterial colonies were mixed with 9.5mL TE buffer, 100μl proteinase K (20mg/mL) and 1mL 10% SDS and incubated for 1 hour at 37°C. 1.8mL of 5M NaCl was added, mixed, and incubated at 65°C for 10 min. Then 1.5mL of 10% CTAB/ 5M NaCl was added, mixed, and incubated at 65°C for 20 min. An equal volume of chloroform iso amyl 24:1 was added, mixed, and rotated on a tumbler for 1 hr. Following centrifugation at 9,320RCF for 10 minutes the top layer was transferred to a clean tube and 5ml of isopropanol was added and mixed. Visible DNA was removed and washed in 500μL 70% ethanol for 5 minutes and dried at 60°C for 10 min. gDNA was resuspended in 500μL – 1mL of molecular grade water.

*Qiagen based gDNA extraction*

For GNB, overnight cultures in LB were centrifuged at 14,000RCF for 10 min. For Staphylococci, the pellet was mixed in 180μl of 20mg/mL lysozyme and 200μg/mL lysostaphin; 20mM Tris HCl, pH 8.0; 2mM EDTA; 1.2% Triton and incubated for 45 minutes at 37°C. For Streptococci, overnight cultures were grown in Todd Hewitt selective medium, centrifuged for 1 minute at 17,000RCF and the pellet subject to bead beating for 10 min. GNB and GPB bacterial isolates gDNA was extracted using the QIAmp DNA mini kit (Qiagen, Germany), with an additional RNAse step, on the automated QIAcube platform (Qiagen, Germany). For mollicutes, *Mycoplasma hominis* was cultured in 30mL of Mycoplasma selective medium and *Ureaplasma spp*. were cultured in 300mL of Ureaplasma selective medium (Mycoplasma Experience ltd, UK), incubated for 4 days at 37°C. Mollicute cultures were centrifuged at 13,000RCF for 3 hrs and resuspended in 400μl of sterile water and extracted using the EZ1 DSP virus kit on the Qiagen EZ1 Advance XL (Qiagen, Germany).

gDNA was quantified using the dsDNA BR assay kit on a Qubit fluorometer 3.0 or 4.0 and kept at 4-8°C or -20°C for chloroform precipitated gDNA and Qiagen extracts respectively.

**Whole genome sequencing**

For Illumina MiSeq, genomic libraries were prepared using Nextera XT V2 (Illumina, USA), with bead-based normalisation. Paired-end WGS was performed using the V3 kit. Each sequencing run was multiplexed up to 48 isolates to provide >20 coverage. For Oxford Nanopore Technology (ONT), gDNA (Qiagen) was concentrated and purified at a 1:1 ratio using SPRI beads (Mag-Bind TotalPure, Omega) with 15µL water elution. Genomic libraries were prepared using the Rapid Barcoding Kit (SQK-RBK004; ONT), sequenced on a FLO-MIN106 R9.4 flow cell using a MinION (ONT). Sequencing was performed on single-use MinION flow cells for a running time of 72 hr with default parameters within MinKNOW unless otherwise specified.

We compared yield and LRS metrics between R9 and R10 flow cells. gDNA (n=53) was extracted using the Qiacube, concentrated using SPRI beads and libraries generated using the 96-Rapid Barcoding Kit (SQK-RBK110.96; ONT). gDNA was pooled and divided into 6 aliquots for simultaneous flow cell loading onto three flow cells; two R10 (one on a MinION Mk1B connected to an Intel i7-8750H laptop, another on a MinION Mk1C) and one R9 (Mk1B connected to an Intel i7-6700 desktop computer). Following the initial sequencing period, flow cells were washed (WSH003), QC checked to determine recovery of nanopores, and re-loaded with the remaining aliquots of gDNA (stored at 4°C).

**Bioinformatics analysis**

*Illumina MiSeq sequencing data: QC of input reads*

Several studies have shown that hybrid *de novo* assemblies significantly improve the quality of contiguous chromosome and plasmid assembly (Wick *et al.*, 2017; Lipworth *et al.*, 2020), and therefore this comparison was not the primary aim. Here, we trimmed Illumina barcodes and applied QC trimming (--phred33 -q 25) with Trimgalore (v0.5.0)(Krueger, 2018). Trimmed fastq were input to Unicycler (Wick *et al.*, 2017) (v0.4.7 and v0.4.9) assemblies. Seqfu (v1.3.1) (Telatin, Fariselli and Birolo, 2021) was used to count the number of reads (paired-end aware). Isolates were excluded from the dataset if SR were insufficient for >1x of the genome ((number of reads*length of reads[~270]/length of genome), in addition to insufficient LR. Further exclusions were based on sequencing read and assembly metrics.

*Basecalling fast5 files and demultiplexing*

Basecalling was performed twice for each isolate. Initial basecalling used Guppy (v2.1.3, v3.2.10, v3.6.5, v4.2.5, and v4.5.4)within MinKNOW. Archived FAST5 were re-basecalled using Guppy v5.0.11 and NVIDIA V100 GPUs. Data in the format of 4,000 FAST5 reads per sub-directory (2018 data) were pre-processed with the ont_fast5_api converter single_to_multi_fast5 parameter. All original LR were demultiplexed using Porechop (v0.2.4) (Wick, 2018b). For all re-basecalled LR, Guppy –trim_barcodes parameter was applied.

*QC for Oxford Nanopore Technology sequencing data*

NanoPlot (v1.19.0) (De Coster *et al.*, 2018) was used to generate LRS metrics including N50, number of reads, and mean read quality (Q score). LRS trimming programs Filtlong (v0.2.0) (Wick, 2018a)(filtlong --min_length 1000 --keep_percent 90) and Nanofilt (v2.6.0) (De Coster *et al.*, 2018) (Nanofilt -q 10 --l 1000 --headcrop 50) were used to create different LR assemblies as described below.

*Hybrid assembly: Illumina MiSeq and Oxford Nanopore Technology*

For all 200 isolates, the original LR were assembled with corresponding SR using Unicycler (v0.4.7)(Wick *et al.*, 2017). Repeat Unicycler hybrid assembly using LR generated from Guppy (v5.0.11), trimmed with Filtlong (v0.2.0; filtlong --min_length 1000 --keep_percent 90) was performed for a subset of isolates (n=62/200).

*Long read (LR) assembly comparison*

A subset of isolates (n=25/200) were selected to evaluate differences in basecalling and *de novo* assembly quality, with LR assemblers compared to hybrid assembly as outlined in Fig 1 and Fig 2. For continuity, isolates included in this analysis were also selected for additional Unicycler hybrid assembly with the latest basecalled LR using Guppy (v5.0.11). The combination of sequence read trimming programs (Filtlong (v0.2.0) and Nanofilt (v2.6.0), LR assemblers (Canu (v2.1.1), Flye (v2.8.1), Raven (v1.5.1) and Miniasm (operated within Unicycler v0.4.9)), and LR assembly polishers is listed in Fig 2. Racon (v1.3.1) (Vaser *et al.*, 2017) was not performed on Canu or Flye assemblies before medaka but was performed on Raven and Miniasm (within Unicycler -l parameter) as default. Medaka_consensus was performed on all assemblies. Default parameters were used throughout except relaxed minimum coverage as required for assembly comparisons. For Miniasm within Unicycler, the --mode conservative was used.

Quast (v5.0.2)(Gurevich *et al.*, 2013), ABRicate (v0.9.7)(Seemann, 2019a) and Mlst (v2.17.6)(Seemann, 2019b) were used to assess assembly quality, identify ARG, and sequence type (ST) respectively. Assemblies were annotated using Prokka (v1.15.5)(Seemann, 2014). Bandage (v0.8.1)(Wick *et al.*, 2015) was used to extract the contig/plasmid of interest and examined for sequence similarity and annotation in Geneious (2020 v1.2). Mash genome distance estimation (v2.3)(Ondov *et al.*, 2016) was performed on all assemblies from the same isolate using the hybrid assembly as reference.

*R9 v R10 sequence read analysis*

Six FAST5 directories (2 per flow cell) were uploaded to a HPC for GPU basecalling using Guppy v5.0.11. Basecalling was performed per flow cell generating three sets of reads per isolate. Nanoplot (v1.19.0) was performed individually and on the concatenation of the reads to one fastq file.

**Cost analysis and computing requirements**

Our cost analysis incorporates pricing from (a) a centralized catalogue incorporating over 80 UK/EU suppliers with Higher Education pricing agreements (Cardiff University, 2021), (b) LMIC-specific pricing where suppliers and manufacturers were willing to negotiate on this point, and (c) not-for-profit research pricing applied to our laboratories’ online catalogue accounts. Start-up costs were calculated for equipment and consumables capable of generating 48 isolates, the least common denominator among kits. Staffing, electrical, shipping, and waste disposal costs were excluded as being too variable between LMIC collaborators to incorporate in this analysis.
