# Supplementary material for: Comparing Long-Read Assemblers to Explore the Potential of a Sustainable Low-Cost, Low-Infrastructure Approach to Sequence Antimicrobial Resistant Bacteria With Oxford Nanopore Sequencing
Source: Front Microbiol. 2022 Mar 3;13:796465. doi: 10.3389/fmicb.2022.796465 (PMC8928191; doi:10.3389/fmicb.2022.796465)
Supplement: Supplementary file 1 [file Data_Sheet_1.zip › SupplementaryFigures.pptx]

## Slide 1
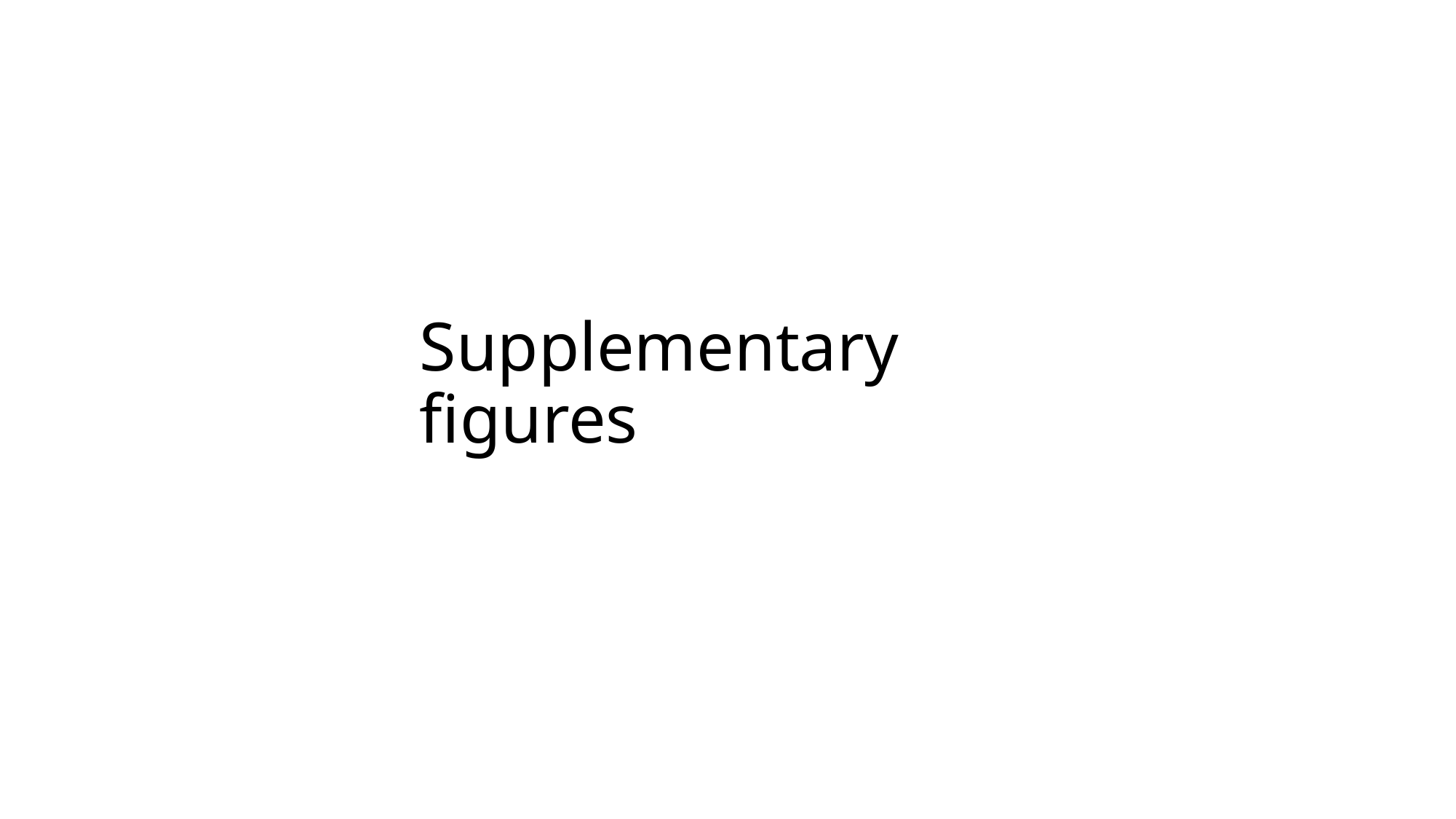

# Supplementary figures

## Slide 2
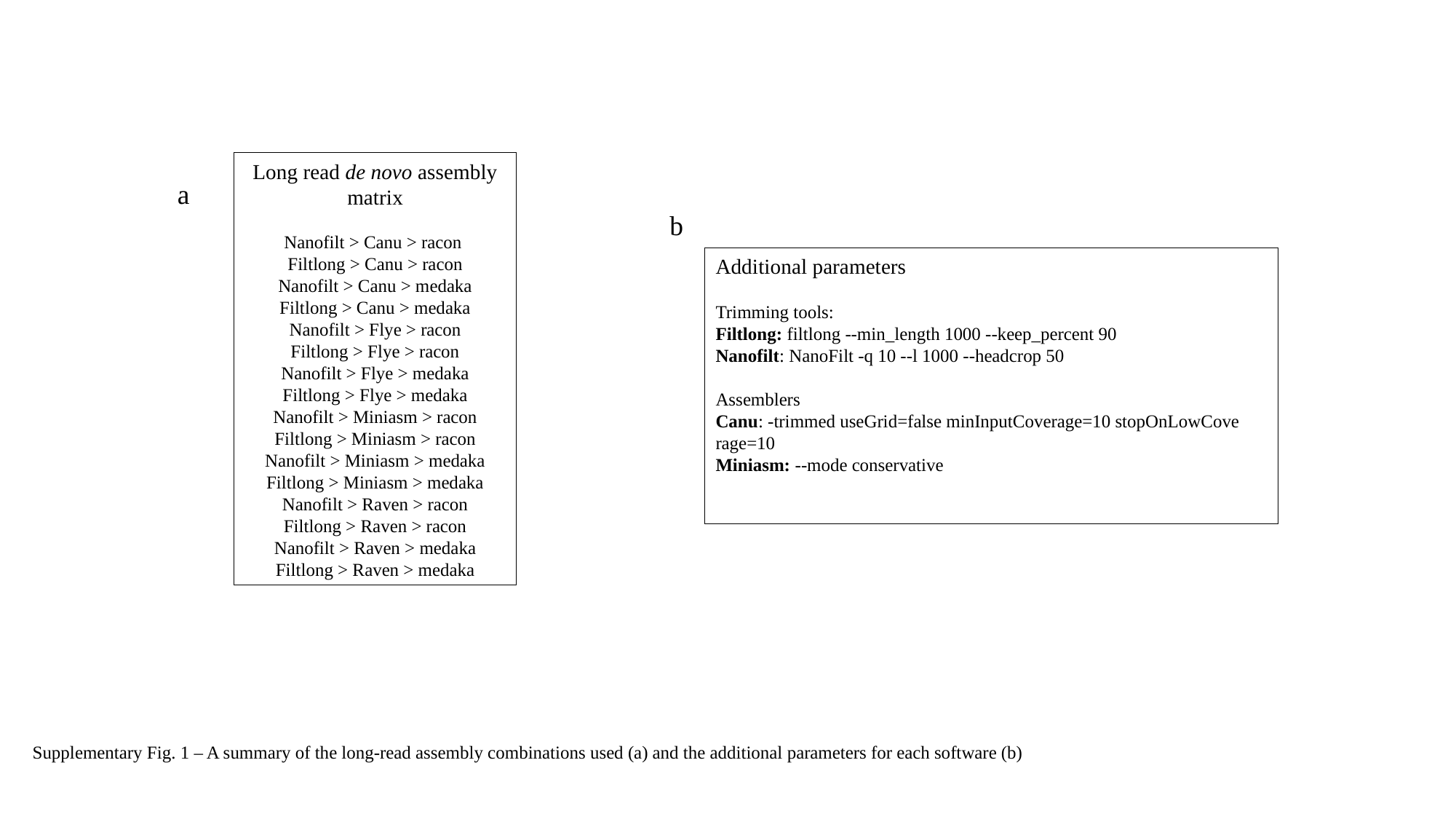

Long read de novo assembly matrix
Nanofilt > Canu > racon
Filtlong > Canu > racon
Nanofilt > Canu > medaka
Filtlong > Canu > medaka
Nanofilt > Flye > racon
Filtlong > Flye > racon
Nanofilt > Flye > medaka
Filtlong > Flye > medaka
Nanofilt > Miniasm > racon
Filtlong > Miniasm > racon
Nanofilt > Miniasm > medaka
Filtlong > Miniasm > medaka
Nanofilt > Raven > racon
Filtlong > Raven > racon
Nanofilt > Raven > medaka
Filtlong > Raven > medaka
a
b
Additional parameters
Trimming tools:
Filtlong: filtlong --min_length 1000 --keep_percent 90
Nanofilt: NanoFilt -q 10 --l 1000 --headcrop 50
Assemblers
Canu: -trimmed useGrid=false minInputCoverage=10 stopOnLowCove
rage=10
Miniasm: --mode conservative
Supplementary Fig. 1 – A summary of the long-read assembly combinations used (a) and the additional parameters for each software (b)

## Slide 3
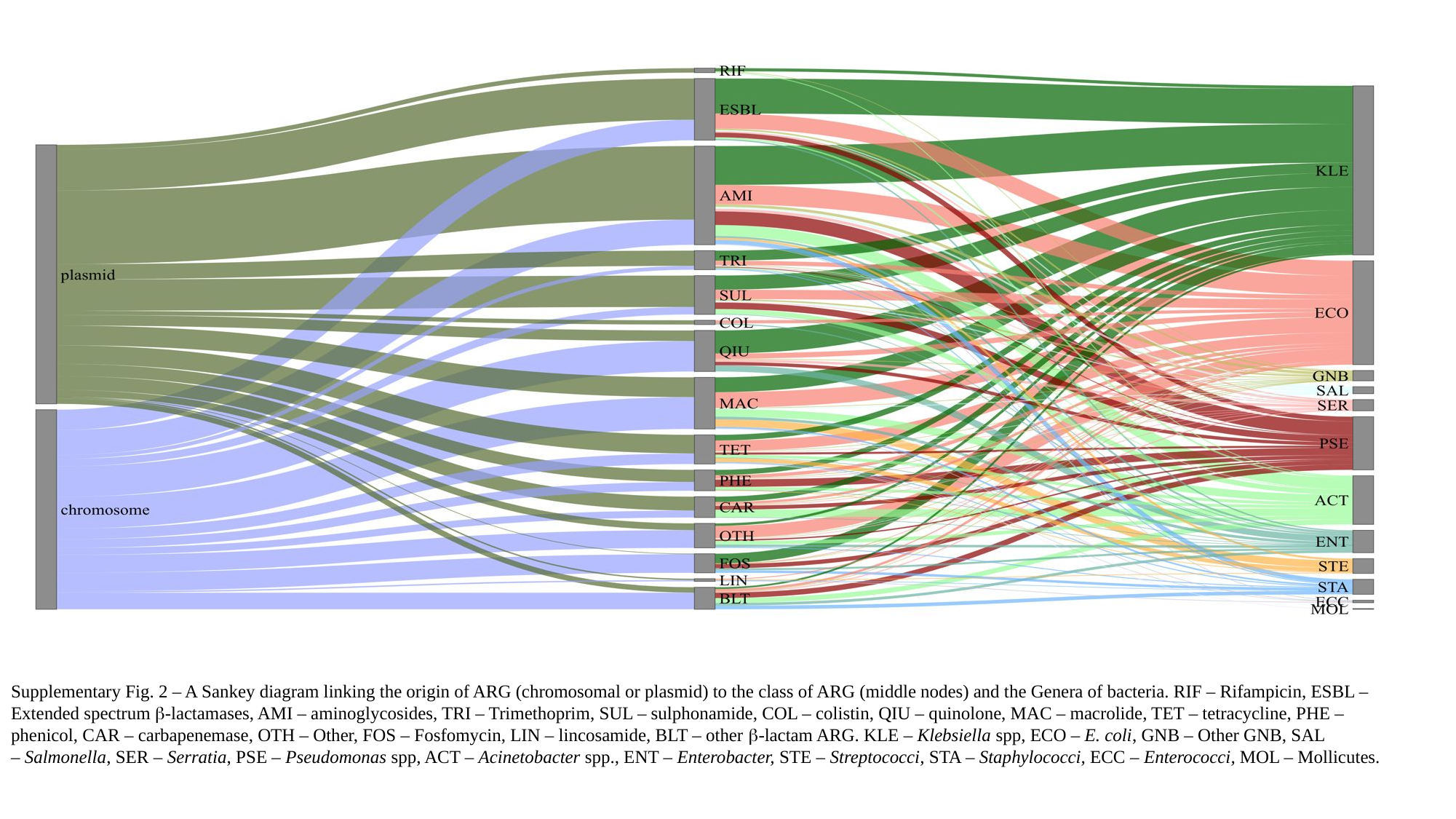

Supplementary Fig. 2 – A Sankey diagram linking the origin of ARG (chromosomal or plasmid) to the class of ARG (middle nodes) and the Genera of bacteria. RIF – Rifampicin, ESBL – Extended spectrum b-lactamases, AMI – aminoglycosides, TRI – Trimethoprim, SUL – sulphonamide, COL – colistin, QIU – quinolone, MAC – macrolide, TET – tetracycline, PHE – phenicol, CAR – carbapenemase, OTH – Other, FOS – Fosfomycin, LIN – lincosamide, BLT – other b-lactam ARG. KLE – Klebsiella spp, ECO – E. coli, GNB – Other GNB, SAL – Salmonella, SER – Serratia, PSE – Pseudomonas spp, ACT – Acinetobacter spp., ENT – Enterobacter, STE – Streptococci, STA – Staphylococci, ECC – Enterococci, MOL – Mollicutes.
